# Structure of outer membrane protein G in lipid bilayers

**DOI:** 10.1038/s41467-017-02228-2

**Published:** 2017-12-12

**Authors:** Joren S. Retel, Andrew J. Nieuwkoop, Matthias Hiller, Victoria A. Higman, Emeline Barbet-Massin, Jan Stanek, Loren B. Andreas, W. Trent Franks, Barth-Jan van Rossum, Kutti R. Vinothkumar, Lieselotte Handel, Gregorio Giuseppe de Palma, Benjamin Bardiaux, Guido Pintacuda, Lyndon Emsley, Werner Kühlbrandt, Hartmut Oschkinat

**Affiliations:** 1 Leibniz-Institut für Molekulare Pharmakologie, Robert-Rössle-Strasse 10, 13125 Berlin, Germany; 2 Centre de RMN à Très Hauts Champs, Institute des Sciences Analytiques (CNRS, ENS Lyon, UCB Lyon 1), Université de Lyon, 69100 Villeurbanne, France; 3 Max-Planck-Institut für Biophysik, Max-Von-Laue-Strasse 3, 60438 Frankfurt am Main, Germany; 4 Unité de Bioinformatique Structurale, CNRS UMR 3528, Institut Pasteur, 75015 Paris, France; 5 Institut des Sciences et Ingénierie Chimiques, Ecole Polytechnique Fédérale de Lausanne, CH-1015 Lausanne, Switzerland

## Abstract

β-barrel proteins mediate nutrient uptake in bacteria and serve vital functions in cell signaling and adhesion. For the 14-strand outer membrane protein G of *Escherichia coli*, opening and closing is pH-dependent. Different roles of the extracellular loops in this process were proposed, and X-ray and solution NMR studies were divergent. Here, we report the structure of outer membrane protein G investigated in bilayers of *E. coli* lipid extracts by magic-angle-spinning NMR. In total, 1847 inter-residue ^1^H–^1^H and ^13^C–^13^C distance restraints, 256 torsion angles, but no hydrogen bond restraints are used to calculate the structure. The length of β-strands is found to vary beyond the membrane boundary, with strands 6–8 being the longest and the extracellular loops 3 and 4 well ordered. The site of barrel closure at strands 1 and 14 is more disordered than most remaining strands, with the flexibility decreasing toward loops 3 and 4. Loop 4 presents a well-defined helix.

## Abstract (web summary)

Porins, like OmpG, are embedded in the outer membrane of bacteria and facilitate uptake and secretion of nutrients and ions. Here the authors present a protocol for solid state NMR structure determination of proteins larger than 25 kDa and use it to structurally characterize membrane embedded OmpG.

## Introduction

β-barrel membrane proteins perform a host of different functions on the surface of bacteria, mitochondria, and chloroplasts by acting as enzymes, transporters, and/or receptors^1,2^. The 34 kDa outer membrane protein G (OmpG) of *Escherichia coli (E. coli)* ^3,4^ belongs to the subclass of porins, which allow the passive yet selective uptake and secretion of nutrients, ions, and proteins in Gram-negative bacteria. Such porins have short turns on the periplasmic side and long loops on the extracellular side^2^, with the latter potentially being relevant for opening and closing of the pore.

OmpG was discovered following the deletion of genes coding for LamB and OmpF, the main porins for the uptake of sugars in *E. coli*. After a selection procedure to generate phenotypes able to grow on a maltodextrin medium, mutations were found that caused expression of the otherwise silent *ompG* gene^4^. Further biochemical analysis showed that OmpG is able to import mono-, di-, and trisaccharides^3^. The *ompG* gene codes for 301 amino acids of which the first 21 are a signal sequence that is cleaved off upon transition to the periplasm^4^. No evidence of OmpG oligomers was found by native/denaturing polyacrylamide gel electrophoresis (PAGE) analysis or cross-linking experiments, indicating OmpG is a native, functional monomer^4^. Further evidence from electrophysiology studies confirmed the monomeric nature of OmpG^5^.

Previous structural studies by protein crystallography or solution NMR revealed a 14-stranded β-barrel^6–8^. In the crystal structures, the strands constituting the barrel extend much further on the extracellular side than expected, far beyond the ring of outward facing tryptophans and tyrosines that are a hallmark of porins, defining the membrane interface. Yildiz et al.^8^ suggested a pH-dependent opening and closing mechanism. A crystal structure obtained at pH 5.6 (2IWW) shows a closed conformation for the porin, with loop 6 folded into the barrel forming a lid, whereas a structure at pH 7.5 is in an open conformation (2IWV). Based on the observation that two histidines of opposite strands (H231 and H261) are connected by a hydrogen bond in the closed form, Yildiz et al.^8^ proposed a mechanism for pH gating. A crystal structure by Subbarao and van den Berg^7^ at pH 5.5 misses part of the residues in loop 6 (219–230) but otherwise resembles the pH 7.5 structure of Yildiz et al.^8^ Along these lines, solution NMR studies performed at pH 6.3 on protein in dodecylphosphocholine (DPC) micelles^6^ yielded a structure where the length of the β-strands match the probable thickness of the outer membrane of *E. coli* (around 27 Å, corresponding to around 10 residues to cross the membrane)^9^. The entire loop 6 and parts of loop 7 could not be assigned, and almost no long-range restraints could be found for most of the extracellular loops, indicating motional processes and structural heterogeneity. Motion of the extracellular loops was confirmed by heteronuclear nuclear Overhauser-effect spectroscopy (NOESY) experiments^6^. pH gating was also investigated by the group of Essen, who constructed OmpG variants with deleted loops^10^. Those structurally intact porins (4CTD) were still opening and closing in a pH-dependent manner. Conlan et al.^5^ revisited the situation by electrophysiology, demonstrating stochastic behavior in the pH range between 5 and 6.

Here, we determine the structure and dynamics of OmpG embedded in bilayers of *E. coli* lipid extracts, to contribute to the analysis of the observed structural differences and to elucidate functional aspects such as pH gating. We purified the protein in detergent solution and reconstituted it into liposomes created with *E. coli* lipid extracts, which were dialyzed extensively on flat membranes to obtain extended arrays of two-dimensional (2D) crystals. The 2D crystals were investigated by a multi-faceted solid-state magic-angle-spinning (MAS) NMR methodology, including proton detection on ^2^H, ^13^C, and ^15^N-labeled samples under fast spinning conditions, and ^13^C-detected experiments on amino-acid-type selectively labeled samples. This approach utilized the best features of each type of experiment, with proton-detected experiments providing well-resolved backbone correlations and carbon-detected spectra helping to observe entire side chains at reduced overlap and thus more confidently determine the amino-acid type. An additional advantage of using both protonated and deuterated samples was that both amide ^1^H–^1^H restraints from ^1^H-detected experiments, and ^13^C–^13^C restraints from ^13^C-detected experiments could be used jointly during the structure calculation.

As a result, a well-defined structure of OmpG in lipid bilayers is obtained that is more reminiscent of the solution NMR structure than that determined by X-ray crystallography. The extracellular loops show different degrees of flexibility, with loops 3 and 4 well defined and strands 1 and 14 varying much stronger. The utilization of ^1^H–^1^H and ^13^C–^13^C restraints in parallel yields a structure determination protocol that allows for proper definition of helix in loop 4.

## Results

### Assignments

**Fig. 1:**
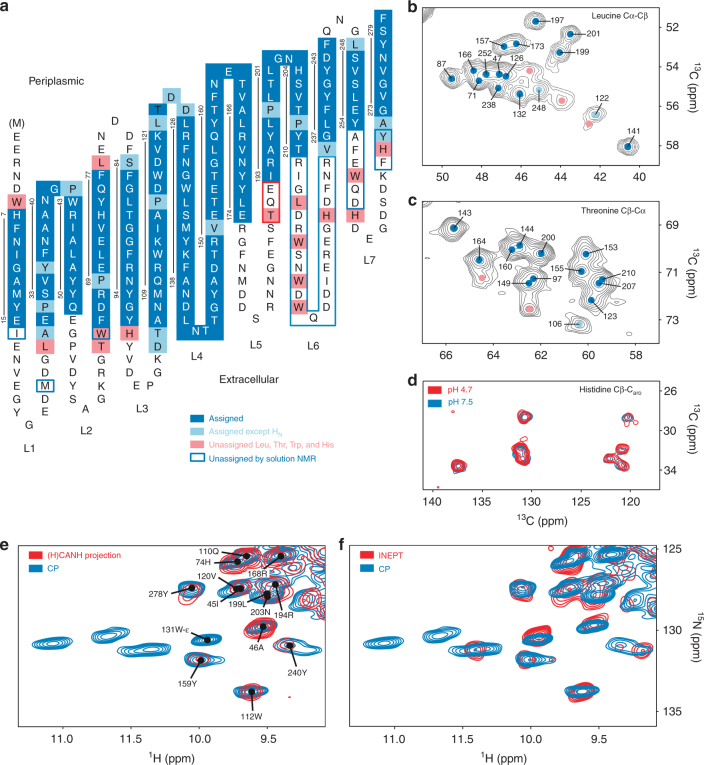
Resonance assignment and OmpG topology. Assigned residues are indicated in blue. **a** For residues in light blue, the ^1^H_N_ shift is unknown but partial carbon assignment was obtained. Pink indicates unassigned residues as discussed in the text. Residues in blue frames do not show signals in solution NMR spectra and residues in the red frame were assigned by solution NMR but not solid-state NMR, see text. Vertical lines indicate the β-strands with residue numbers. **b**–**d** Spectral regions of ^13^C–^13^C correlation spectra comprising Cα–Cβ peaks of **b** leucine in the GAVLS(W) sample (20 ms DARR), **c** threonine in a DARR spectrum of the 1,3-TEMPQANDSG sample (50 ms mixing), and **d** histidine in a 50 ms DARR spectrum of the GANDSH(LV) sample. For the peaks indicated by pink dots in these ^13^C–^13^C spectra, no strip could be found in the ^1^H-detected 3D spectra. **e**, **f** Overlays of a CP-based ^1^H–^15^N-correlation (blue) comprising the region of Trp side chain cross peaks with the projection of the CANH spectrum (**e**) and an INEPT-based HSQC (**f**)

2D-crystalline samples of OmpG were prepared utilizing *E. coli* lipid extracts, and crosschecked by electron microscopy (Supplementary Fig. 1). In order to obtain sequence-specific chemical shift assignments, ^1^H-detected (H)CANH, (HCO)CA(CO)NH, (H)CONH, (H)CO(CA)NH, (HCA)CB(CA)NH, and (HCA)CB(CACO)NH spectra of ^2^H, ^13^C, ^15^N-labeled OmpG with the exchangeable sites protonated to either 100 or 70% were recorded at 60 kHz MAS^11,12^. They were evaluated together with ^13^C–^13^C correlations obtained on amino-acid-type selectively ^13^C-labeled samples, such as GAVLS, GAF_α,β_Y_α,β_, etc. (Table 1). This set included samples prepared by a reverse labeling strategy in which a subset of amino acids, either produced through the glycolysis pathway (SHLYGWAFV) or the citric acid cycle plus glycine, alanine, and serine (TEMPQANDSG) are labeled with the glycerol-derived patterns through feeding the bacteria with [2-^13^C]- or [1,3-^13^C]-glycerol. The respective samples are called henceforth 2- or 1,3-glycerol or simply 2- or 1,3-OmpG, indicating also labeled amino acids^13^. In total, 10 amino-acid-type selective labeling schemes were employed. The combined evaluation yielded the sequence-specific assignment of 170 residues (Fig. 1a; Supplementary Figs. 2, 3) corresponding to 60% of the OmpG sequence (Supplementary Table 1). Of these, for 16 residues, including 6 prolines, only ^13^CA, ^13^CB, and ^13^CO chemical shifts were assigned based on correlations to the assigned HN resonances of the following residues in the (HCO)CA(CO)NH, (H)CONH, and (HCA)CB(CACO)NH spectra. For three assigned residues, only signals in the ^13^C-detected spectra were observed. The proton-detected (H)CANH contained 182 cross peaks (Supplementary Fig. 2), of which 31 remained unassigned. During this assignment process, amino-acid types were determined or verified by CA, CB, and side chain ^13^C chemical shifts, as derived by inspection of the 2D ^13^C–^13^C dipolar-assisted rotational resonance (DARR) spectra recorded on the amino-acid selectively labeled samples (e.g., Fig. 1b, c), taking into account isotope shifts in the deuterated sample^14–20^.

**Table 1:** Amino acid-type selectively ^13^C-labeled OmpG samples produced for sequence-specific assignments and distance measurements

Residue specific	[2-^13^C]- or [1,3-^13^C]-glycerol
GAF_α,β_Y_α,β_ (S)	2- and 1,3-uniform
GAVLS(W_α,β,γ_)	2- and 1,3-TEMPQANDSG
RIGA(S)	2-SHLYGWAFV(QENDT)
GANDSH(LV)	1,3-MKINDT
GENDQPASR	
GAF_α,β_Y_α,β_ SHVL	

Amino acids in brackets were accidentally labeled to a lower degree due to active biochemical pathways. Samples in the left column were prepared by adding ^13^C, ^15^N-labeled amino acids (or as specified) to ^15^NH_4_Cl-containing growth medium so all others appeared ^15^N- but not ^13^C-labeled. Samples in the right column were prepared by a “reverse” labeling scheme in which either [2-^13^C]- or [1,3-^13^C]-glycerol medium was used to produce the respective ^13^C-labeling pattern for the indicated amino acids, whereas all other amino acids were added in ^15^N-labeled form to the growth medium

For most amino acids, there is at least one spectrum obtained on the amino-acid specifically labeled samples where the intra-residue Cα–Cβ peaks are resolved and the type of amino acid can be identified or the possibilities can be substantially limited. Additional side chain ^13^C chemical shifts beyond Cβ are also accessible, further reducing the ambiguities occurring during the sequential assignment procedure. For this purpose, due to better signal-to-noise and longer mixing times enhancing long-range transfers through side chains, the 2D ^13^C–^13^C spectra were often more useful than the ^13^C-detected three-dimensional (3D) spectra (NCACX, NCOCX). The resulting assignments are shown in Fig. 1, with the assigned residues indicated in dark blue when the NH groups and backbone carbon atoms as well as Cβ were assigned, and in light blue when an amide proton could not be detected. In total, 111 residues remained unassigned due to the lack of sufficiently intense signals in the proton- or carbon-detected spectra. Assignments can be found in the BMRB (ID 34088) and are indicated on the CA-N projection of the (H)CANH experiment (Supplementary Fig. 2a).

As noted earlier, of the 281 residues we observed 182 cross peaks in the (H)CANH spectrum, of which 151 were unambiguously assigned. For most of the other 31 peaks, the signal-to-noise ratio was very low hence no sequential correlations were found in the less sensitive 3D spectra. A comparison of the cross polarization (CP)-based 2D ^1^H–^15^N spectrum with the projection of the (H)CANH shows many small, unassigned peaks in the 2D correlation, located in a region indicative of random coil secondary structure (Supplementary Fig. 2a). Incomplete back-exchange of ^1^H at amide positions can be excluded as a reason for unobservable or weak resonances since the protein was purified under denaturing conditions and refolded. In addition, most of the weak signals arise from residues in the loop regions, see Fig. 1, whereas the transmembrane region is assigned, indicating efficient back-exchange.

We rather attribute the low-signal intensity or absence of signals to mobility and/or structural heterogeneity. Motion adversely affects the efficiency of cross polarization, which lowers signal intensity in solid-state MAS NMR spectra. Structural heterogeneity with slow transitions (on the NMR timescale) between states leads to a splitting or distribution of signals and hence to signal broadening that reduces signal-to-noise. To analyze the situation regarding dynamics and structural heterogeneity closer, we inspected intensities and line shapes of cross peaks in suitable regions of the 2D ^13^C–^13^C spectra. Leucine and threonine Cβ–Cα cross peaks of assigned residues (Fig. 1b, c, dark blue dots) appear strong, e.g., with symmetrical line shapes. The light blue dots indicate carbon signals of residues for which no signal of the NH pair was found. For the pink-labeled cross peaks no assignments were possible. Those cross peaks are of lower intensity, and some of the line shapes reveal considerable heterogeneous broadening. The unassigned leucine and threonine residues (pink in Fig. 1a) cluster near the transmembrane region of the protein in the extracellular loops or intracellular turns, one to three residues away from the last assigned residue. Other residue types exhibit a more pronounced difference: in a sample containing ^13^C-labeled histidine but no other aromatic residues in labeled form, only 4 of 7 expected signal sets are observed (Fig. 1d) of which 3 were assigned (H7, H74, H204). Tryptophan residues are also good reporters since their side chain NH signals may be easily observed in ^1^H–^15^N correlation spectra and distinguished from other signals. Four tryptophan residues are assigned. Of the unassigned Trp residues, two are located very close to assigned residues, while the remaining four are in loop 6 and 7 (pink residues in Fig. 1a). When comparing a (H)CANH projection with the CP-based HSQC (heteronuclear single quantum coherence) spectrum, only side chain signals of five tryptophan residues are identified (Fig. 1e; Supplementary Fig. 2a). The insensitive nuclei-enhanced by polarization transfer- (INEPT) based HSQC spectrum does not show additional signals, contrary to what is often observed for flexible residues (Fig. 1f; Supplementary Fig. 4). We conclude that some of the tryptophan and histidine residues in loop 6 and 7 do not show signals; they are missing even in the more sensitive 2D correlation spectra. We further inspected the cross-peak in the (H)CANH, (HCO)CA(CO)NH, (HCA)CB(CA)NH, and (HCA)CB(CACO)NH spectra and plotted their intensity vs. the sequence (Supplementary Fig. 5), noting that intensities decrease toward the ends of the strands. The decrease of signal intensity toward the bilayer boundaries indicates an increase in motional processes for residues closer to the surface. Together with the results from the analysis of the 2D spectra, motional processes are considered as main reasons for the lack of loop signals.

The dynamics of the loops could potentially be affected by pH-dependent opening and closing of the porin. It was first proposed to depend on interactions between two histidine residues, H231 and H261^8^. In order to investigate this situation further and to test whether the residues with missing signals become more ordered or rigid upon pH change, we compared spectra recorded around neutral pH and at pH 4.7 on samples with labeled G, A, L, V, S, H, F_α,β_, and Y_α,β_. Both spectra showed a very similar signal pattern overall, and in particular in the aromatic region (Fig. 1d), where only four histidine signal sets were observed. Lowering the pH did not reveal additional histidine signals, as would be expected if loops 6 and 7 became more structured or more flexible.

This situation did not change substantially upon cooling, a strategy employed to decrease motions which may be interfering with averaging by MAS and thus obscuring signals. In spectra recorded at 255 and 235 K 1D cross polarization efficiency did not differ significantly and very similar 2D ^13^C–^13^C fingerprint spectra were observed, with perhaps more signals in the spectra obtained at the higher temperature as opposed to the converse (Supplementary Fig. 6).

### Structure calculations

Distance restraints were collected from both the ^1^H- and ^13^C-detected experiments to provide a protocol that is independent of secondary structure. In particular, restraints between amide protons are valuable for defining β-sheet topology, whereas carbon–carbon restraints are instrumental for defining α-helical structures. Because the structure calculations were performed employing automated ambiguous distance restraints, the cross peaks were carefully analyzed to ensure peaks from unassigned residues do not appear in spectra delivering distance-dependent information, as described in the previous section. While the ^1^H and ^13^C data used for restraints were acquired with sample temperatures of around 300 and 280 K respectively, other ^13^C-detected data have been acquired at various temperatures ranging from 300 K to below 260 K, however, no substantial changes were observed in ^13^C–^13^C or ^15^N–^13^C correlations acquired over this range.

**Fig. 2:**
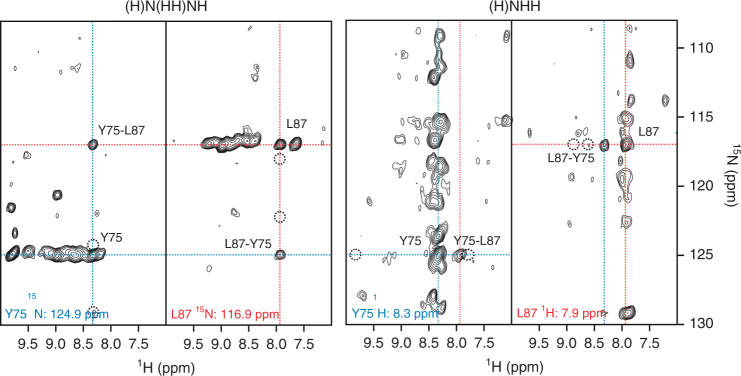
Set of two planes from the 3D (H)NHH and (H)N(HH)NH spectra. Strips taken at the ^15^N chemical shifts of Y75 (left) and L87 (right) from the (H)N(HH)NH and (H)NHH spectra, respectively. The proton–proton cross-peak pattern is indicative of cross-strand hydrogen bonding between the backbone amide and carbonyl groups of tyrosine 75 and leucine 87. Red lines correspond to the ^1^H and ^15^N chemical shifts of L87. Blue lines correspond to the ^1^H and ^15^N chemical shifts of Y75. A total of four cross peaks are present at the intersections of red and blue lines. Dotted circles indicate positions of potential sequential cross peaks (see text)

A pair of 3D (H)NHH and (H)N(HH)NH spectra with 2 ms radio frequency-driven recoupling (RFDR) mixing^21^ were acquired on the perdeuterated sample, where the exchangeable sites contained protons close to 100%, yielding 249 through-space amide–amide cross peaks (Supplementary Table 2). For each residue, the spectra showed an auto-correlation peak along with one large and often one or two smaller cross peaks. In the case of an ideal anti-parallel β-sheet, those strong off-diagonal peaks are due to interactions of protons from hydrogen-bonded amide groups that face each other from neighboring strands at a distance of 3.3Å. The smaller peaks are usually correlations to the amide groups of sequentially neighboring residues (4.3Å in an ideal β-strand). If both spectra are evaluated side by side, four large cross peaks can be found, indicating the spatial proximity of two amide groups. Figure 2 shows a set of two planes from the two 3D spectra, taken at the ^15^N or ^1^H chemical shifts of Y75 and L87. The strong cross-strand peaks are indicated by cross-hairs. The locations of the expected sequential cross peaks are indicated by circles. The RFDR mixing time of 2 ms was chosen to be relatively short, to favor the short cross-strand distance relative to the correlations between more distant, sequential protons. Ambiguous distance restraints (ADRs) were produced by automatically matching assigned chemical shifts with the RFDR peak lists.

A total of 1847 peaks were identified in 11 2D ^13^C–^13^C correlation spectra of the 2- and 1,3-glycerol (200 and 400 ms DARR), 2- and 1,3-TEMPQANDSG (150 and 400 ms DARR), 2-SHLYGWAFV (150 and 400 ms DARR), and GAF_α,β_Y_α,β_ (500 ms DARR) samples, see Supplementary Table 2. Only peaks in the aliphatic region of the spectra were selected since the chemical shift assignment for this region is relatively complete. Examples are given in Supplementary Figs. 7 and 8. Also, intra-residue peaks were excluded to prevent the automatic chemical shift-matching procedure from generating faulty ADRs based on unassigned intra-residue peaks, for which the correct assignment option is missing. Such intra-residue peaks were identified by comparison of the spectra recorded with short and long mixing times. Assignment possibilities for the ADRs were reduced via a CCPNMR analysis tool that explicitly considers labeling schemes and were limited to pairs of carbon spins for which the product of the labeling percentages exceeded 10%.

About 128 φ/ψ torsion angles (256 in total) were predicted using the program TALOS+^22,23^. As expected, the vast majority of assigned residues are predicted to be in a β-sheet conformation (Supplementary Fig. 9). These results are in good agreement with a prediction of the topology based solely on the amino-acid sequence by the program PRED-TMBB, which is specifically designed for the topology prediction of transmembrane β-barrels (Supplementary Fig. 9, bottom row)^24^.

Structures were calculated without explicit, manual assignment of distance restraints by a modified ambiguous restraints for iterative assignment (ARIA) protocol^25,26^, making a stepwise use of data from proton- and carbon-detected experiments. ^1^H-detected restraints between amide protons are very appropriate for constraining the backbone conformation of a protein that is almost entirely β-sheet. Therefore, in the first four iterations of the protocol, these were the only distance restraints employed (Supplementary Fig. 10). After the first iteration, the lowest-energy structures clearly show the shape of a β-barrel (Supplementary Fig. 13). Starting with the fifth iteration, the more ambiguous ^13^C–^13^C distance restraints were added. ADRs that did not contribute an assignment option within the distance violation tolerance for at least half of the lowest-energy structures from the previous iteration step were rejected by ARIA’s violation analysis. Supplementary Figures 10–12 show the degree of restraint disambiguation by the ARIA protocol. No hydrogen bond restraints were added in those initial structure calculations, yielding an initial structural bundle with a pairwise backbone root mean square deviation (rmsd) of 2.06 ± 0.42Å for residues in the β-sheet (Supplementary Fig. 13, iteration 8). Guided by this structure, 92 co-linear hydrogen bond restraints were derived for the β-sheet region, 2 for every interacting pair of residues in two adjacent β-strands if the characteristic cross-peak pattern indicating hydrogen bonding was observed in the 3D spectra and TALOS+ results indicated β-sheet secondary structure.

**Fig. 3:**
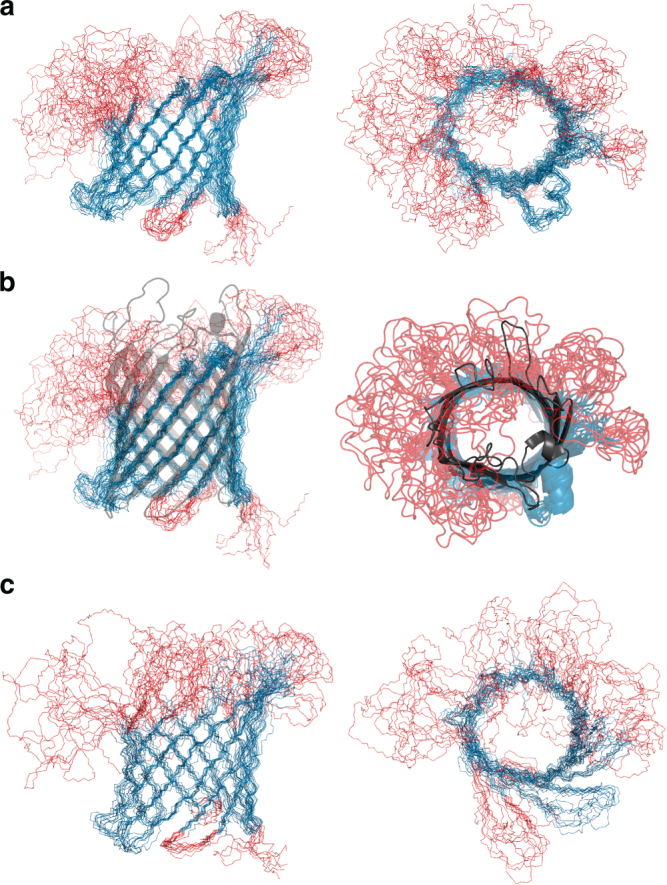
Solid-state NMR structure of OmpG in lipid bilayers and comparison to X-ray and solution NMR structures. **a** Regular secondary structure is shown in blue, loop regions in red. The structures to the right are turned by 90°. **b** Overlay of solid-state (blue and red) and X-ray structure (dark gray). The beta-sheet is extended further in the model derived by X-ray crystallography (2IWV), see left edge. **c** Same views of the solution NMR structure 2JQY obtained from OmpG solutions in dodecylphosphocholine. Figure generated using pymol^53^

The structures calculated with all restraints (Fig. 3a) display a well-defined β-barrel in the membrane-integrated region of the porin, consisting of 14 strands of varying length that span the membrane. On the extracellular side, the strands 5, 6, 7, and 8 extend far beyond the membrane surface, before forming the well-ordered loops 3 and 4. The NMR data reveal that loop 3 and 4 stabilize each other by several interactions. Conversely, the strands preceding loops 1, 2, 6, and 7 on the same side become disordered right after the membrane boundaries. In our structure, these loops adopt many different conformations due to the lack of NMR signals and hence structural restraints (Fig. 1a). The short turns on the intracellular side are mostly well defined. At the top of loop 4, a short α-helix is observed, well defined by a large number of carbon restraints.

### Structure comparison

The solid-state NMR structure is similar to the published X-ray and solution NMR structures (Fig. 3b, c) in the membrane-integrated region of the β-barrel and its periplasmic turns, with an overall rmsd of ~2.0 Å. It deviates from the crystal structures in the extracellular part of the protein. Whereas loops 1, 2, 6, and 7 are found to be flexible by solid-state NMR for OmpG in lipid bilayers, the β-barrel is much more extended in the crystal structures. A comparison is shown in Fig. 3b, with the structure 2IWV aligned with the NMR ensemble. Close inspection of the crystal lattice reveals that the β-sheet is almost entirely continuous from the bottom to the top of the loops, of which loops 3, 4, and 6 are stabilized by a network of crystal contacts (Supplementary Fig. 14a). An interesting picture is obtained when superimposing all available X-ray structures^7,8,10,27,28^ 4CTD (loop 6 deletion), 2IWW, 2IWV, 2P1C, 2X9K, 2WVP (cysteine mutant synthetically modified). In this superposition, loops 3, 4, and 5 adopt very similar positions, and loops 1, 2, 6, and 7 diverge considerably, although much less so than in the NMR structures (Supplementary Fig. 14b).

Conversely, the solid-state NMR structure determined on protein embedded in lipid bilayers is very similar to the solution NMR structure obtained on detergent-solubilized material (Fig. 3c; Supplementary Fig. 14c). The extent of the β-sheet is almost identical. The largest difference between the two structures is indicated in Fig. 1a: between strands 9 and 10 an additional set of NOE cross peaks between two pairs of amide groups could be observed in the liquid state, demonstrating the presence of four extra hydrogen bonds that were added in the calculation of the respective detergent solution structures. In bilayers of *E. coli* lipid extracts, however, the corresponding stretch of residues (Thr190, Gln191, and Glu192) in strand 10 was not assigned. Since the opposing strand was assigned, it was possible to search for cross-strand correlations. However, no cross peaks are present in any of our spectra that could indicate interactions within residue pairs Thr190–Glu174 and Glu192–Tyr172. Thr190 is one of the two unassigned threonines shown in Fig. 1c. Since threonines are in general easy to assign, and because of their distinct chemical shift pattern, it is evident that the signals indicative of hydrogen bonds in this area are absent.

An interesting question concerns the position of the α-helix that is reported by all methods, and that is defined by a large number of carbon distance restraints in our solid-state NMR structure. Here, the helix is situated largely outside of the barrel, nearly perpendicular to the sheet. In the X-ray structures loops 4 and 5 pack against each other, pushing the helix into a position where half of it faces into the pore. The detergent-solution NMR structure (Fig. 3c) shows the helix less defined but the respective region approximately in the same position as in the MAS NMR structure, with a larger spatial distribution due to the lack of side chain restraints (Supplementary Fig. 14c).

## Discussion

A 3D structure of OmpG from *E. coli* in bilayers composed of *E. coli* lipid extracts was determined by MAS NMR spectroscopy in a de novo manner. 2D-crystalline arrays were produced prior to the measurements, and the 2D-crystalline state of each sample was validated by electron microscopy before being packed into rotors (Supplementary Fig. 1). The structure is defined by a large number of proton–proton and carbon–carbon restraints (Supplementary Table 2), showing a well-defined β-barrel for the membrane-integrated region of the structure. On the side of loops 3 and 4, an extended barrel structure is observed, and an α-helix is located on top of loop 4. In contrast, loops 1, 2, 5, 6, and 7 are not well defined, with considerable structural heterogeneity observed in membrane proximal sections, with the signals of the respective residues either weak or not observed in two- and three-dimensional NMR spectra. This contrasts with the consensus X-ray structures, in which the barrel is much longer and consists of a regular, cylindrical β-sheet. However, the superposition of related X-ray structures^7,8,10,27,28^ (Supplementary Fig. 14b) clearly shows that loops 1, 2, 6, and 7 have a degree of conformational flexibility, while loops 3, 4, and 5 look very similar, and are hence more rigid, perhaps due to restraints by interactions within a protomer or in the crystal lattice. This favors an explanation for the structural differences between the X-ray and the solid-state NMR structure that invokes a role of larger conformational freedom associated with loops 1, 2, 6, and 7 in the NMR case. The solid-state NMR structure strongly resembles the detergent-solution NMR structure determined by Liang and Tamm^6^, with the exception of the lone α-helix being better defined. Overall, the NMR and the body of X-ray structures support a consensus, represented by a 14-stranded, membrane-spanning β-sheet, and indicating considerable potential for mobility in loops 1, 2, 6, and 7, whereas loops 3 and 4 appear well ordered. For loop 5, a different picture is obtained in the X-ray and NMR cases, with few divergences in the superposition of X-ray structures but lacking definition in the NMR structures. The increase in loop mobility and thus of the porin structure toward the meeting point of N- and C-terminus is remarkable.

The current study adds to earlier mechanistic investigations as to the pH-dependent opening and closing^10,29,30^. According to our study, the loops remain dynamic at low and neutral pH even when the protein is embedded in lipid bilayers, making it unlikely that a hydrogen bond between histidines 231 and 261 plays a role in closing. Moreover, our experiments at low pH (e.g., Fig 1d) lead to nearly indistinguishable solid-state NMR spectra (within the set of visible signals), indicating that only minor changes in the pore occur. This does not exclude, however, the hypothesis that pH-dependent conformational ensembles in the loops lead to more or less open or closed states as purposed by Zhuang et al., since in contrast to the solution NMR spectra the respective signals are not detected in the solid-state NMR spectra. A selective movement of strands within the membrane was not apparent from the spectra recorded at different pH.

The structure nurtures the speculation that the ordered loops 3 and 4 are docking sites for possible interaction partners while the helix may provide specificity. The reason for the apparent mobility or the structural, static disorder of the other loops remains unclear. Inspection of the cross peaks from unassigned leucine and threonine residues (see above) leads to the conclusion that structural heterogeneity starts in the membrane proximal region, and the lower CP efficiencies suggest considerable mobility.

The structure was determined by a new general protocol that combines data from MAS experiments at very fast spinning rates employing sensitive ^1^H-detection with ^13^C-detected data from experiments on samples ^13^C-labeled in an amino-acid-type selective manner for both resonance assignments and restraints collection. Distance restraint assignment was achieved in an automated manner during structure calculation, without manual interference, using ARIA supported by CCPN^31,32^ and starting from random coordinates. The protocol is robust and enables de novo structure determination of comparably large systems such as demonstrated here for the 180-residue portion of the 280-residue membrane protein OmpG. It ensures a minimum of operator bias while exploiting a large number of medium- and long-range distance restraints (>600). In terms of methodology, it thus adds to earlier structural studies on membrane proteins in a microcrystalline state^33^ and in lipid bilayers^34–36^ by applying a combination of ^1^H- and ^13^C-detected experiments, also making use of amino-acid-type selectively labeled samples, enabling the automated structure determination of a large system and thus proving the robustness of the approach. The combination of data from ^1^H- and ^13^C-detected experiments makes the strategy independent of the topology of the membrane protein. Here, the data from the proton-detected experiments are clearly most important for defining the porin structure, which has predominantly β-sheet topology, whereas in case of an α-helical membrane protein the side chain–side chain contacts required for defining the fold would be accessible from the carbon-detected experiments. As an example, the helix in OmpG was well defined in our solid-state NMR structure due to those carbon–carbon restraints, but less so in the solution NMR structure (Supplementary Fig. 14c). In future, and with new hardware available that enables MAS up to 150 kHz or more, we expect that proton–proton contacts between side chain sites may be measured using non-deuterated protein.

In this paper, we report the structure of the porin OmpG determined by solid-state NMR in lipid bilayers, which is the largest determined in a de novo manner by this method so far. This study serves as a blueprint for structure determination of membrane proteins in lipid bilayers and of large protein complexes. It further emphasizes the potential of solid-state NMR for atomic resolution structure determination when loop conformations in membrane proteins are important to explain function. In this context, current methodological developments such as MAS beyond 110 kHz enabling measurements of ^1^H–^1^H contacts in fully-protonated biomolecules, and dynamic nuclear polarization will increase its reach further.

## Methods

### Preparation of 2D-crystalline samples of OmpG

All OmpG samples were produced using the same principal preparation protocol. For some of the preparations, however, minor modifications were necessary, which are listed in separate subsections below. Overall, the procedure consists of the following steps^37^: (i) the protein was expressed in *E*. *coli* Bl21 (DE3) and appeared in inclusion bodies. (ii) After purification under denaturing conditions, the protein was refolded in a detergent-containing buffer. (iii) Subsequently, the protein was reconstituted into lipid bilayers made up by *E. coli* total lipid extract^38,39^ to form 2D crystals upon dialysis^40^. The crystalline nature of these 2D crystals was checked by electron microscopy (Supplementary Fig. 1).

### Expression of OmpG with ^13^C and ^15^N-labeling schemes

For experiments employing carbon detection, samples with two main labeling schemes were used in this study: (i) uniform, systematic ^13^C, ^15^N labeling, using [*u*-^13^C]-glucose, [1,3-^13^C]-, or [2-^13^C]-glycerol (the resulting samples made with the glycerol isotopologues will be referred to as 1,3-OmpG or 2-OmpG, respectively) as sole carbon source and [^15^N]-NH_4_Cl as sole nitrogen source^18^; (ii) amino-acid-type selective labeling, achieved by applying either “forward” or “reverse” protocols. For forward labeling, a specific set of ^13^C, ^15^N-labeled amino acids was added to the medium, whereas the remaining amino acids were added in unlabeled form, as sole carbon and nitrogen source; for reverse labeling, a subset of amino acids was added in unlabeled form and the ^13^C, ^15^N-labeled amino acids were produced by biosynthesis using media containing [1,3-^13^C]- or [2-^13^C]-glycerol, and [^15^N]-NH_4_Cl as sole nitrogen source^13^. Amino acid-type selective labeling was applied to decrease spectral overlap and to provide complementary information for the sequential assignment process and restraint disambiguation. To be aware of effects of scrambling, metabolic and catabolic pathways were cross checked beforehand, using the ECOCYC database which includes most of the biochemical pathways of *E. coli* K12^41^. The labeling patterns of all preparations were analyzed and verified by recording ^13^C–^13^C proton-driven spin diffusion (PDSD) or DARR spectra. In the sections below, the preparation of individual samples is described, whereby the labeling pattern desired is given in amino acid one-letter code and accidentally labeled amino acids are given in brackets, or according to IUPAC in square brackets.

### Using labeled glycerol as carbon source

An overnight culture was diluted to an optical density of 0.1 (measured at 600 nm) in M9 minimal media containing 2 g L^−1^ of either [1,3-^13^C]- or [2-^13^C]-glycerol as sole carbon source and 0.5 g L^−1^ [^15^N]-NH_4_Cl as sole nitrogen source^18^. At an optical density of 0.6–0.7, the expression of OmpG was induced by isopropyl-β-d-thiogalctopyranoside (IPTG, 1 mM). Cells were further incubated for 3 h at 37 °C and collected by centrifugation at 5.000 × *g* for 15 min at 4 °C. The pellet was washed with ice-cold sodium chloride solution (500 mL, 0.15 mM), centrifuged at 5.000 × *g* for 15 min at 4 °C and the resulting pellet was stored at −80 °C.

### Forward labeling of OmpG

Several samples with different labeling scheme were produced. For the samples with the pattern GAF_α,β_Y_α,β_ (S) and GAF_α,β_Y_α,β_ SHVL, cells are grown first on unlabeled rich media (pre-culture) and then transferred into a small volume of labeled media allowing growth to high-cell densities^42^. The general protocol is as follows^16^: cells were grown in 4 L of Luria Bertani medium (LB medium) at 37 °C while shaking at 180 rpm. Upon reaching optical cell densities of ~0.5 (measured at 600 nm), the cells were pelleted by centrifugation at 5.000 × *g* and 4 °C for 15 min. The cells were then washed and pelleted using a 1× M9 salt solution, to remove all nitrogen and carbon sources. Afterwards, the cell pellet was re-suspended in 2 L of isotopically labeled media containing 200 mg of each labeled and unlabeled amino acid, 2 g of glucose, and 0.5 g of NH_4_Cl per liter of culture and then incubated to allow the recovery of growth and clearance of unlabeled metabolites. Protein expression was induced after 1 h by the addition of IPTG. After a 4 h incubation period, the cells were harvested and stored at −80 °C.

The samples with the pattern GAVLS(W_α,β_,Cʹ), RIGA(S), and GANDSH(LV) were produced by high-cell density fermentation^43^. The fermentation procedure comprises the following steps: batch phase growth of cells; fed phase in which the culture is grown to high-cell densities; adaptation and expression phase after switching to a labeled feed. For adaptation and expression, a separate amino-acid feed was applied in which 130 mg of each amino acid, labeled or unlabeled (except tyrosine: 100 mg), was dissolved in 140 ml of 2× M9 salt solution. At the beginning of the expression phase, 35 ml of the amino-acid feed was manually added. After 30 min, expression was induced by the addition of 1 mM isopropylthio-β-d-galactoside (IPTG, 5 ml of a 1 mM solution). The remainder amino-acid feed was pumped into the medium at a rate of 30 mL h^−1^. Cells were harvested and stored at −80 °C after 3.5 h of expression. All other preparation steps were done as described before^37^. In 2D ^13^C–^13^C DARR spectra of the GANDSH(LV)-OmpG sample considerable scrambling was observed, which we attribute to anabolic or catabolic enzymatic reactions involving precursors of the amino acids Q, E, D, and N.

### Forward labeling of GENDQPASR-OmpG

To avoid scrambling as observed for the GANDSH(LV)-OmpG sample, we used a protocol in which the enzymes of the anabolic or catabolic reactions connected to the amino acids Q, E, D, and N were blocked by using specific inhibitors^44^. The protocol is in principle following the procedure described above for the preparation of the GAF_α,β_Y_α,β_ (S) and GAF_α,β_Y_α,β_ SHVL-OmpG samples.

The pellet of the pre-culture was re-suspended into M9 minimal media containing unlabeled amino acids (H, F, Y, C, K, L, M, T, I, W, and V, each 1.0 g L^−1^) and labeled amino acids (G, N, D, Q, R, E, P, A, and S, each 0.1 g L^−1^). Additionally, inhibitors were added using the following concentration: 180 mg L^−1^ of l-methionine sulfone, 45 mg L^−1^ of sodium succinate, 45 mg L^−1^ of sodium maleate, 45 mg L^−1^ of aminoxy acetate, and 45 mg L^−1^ of dl-malate. Protein expression was induced after 15 min by the addition of 1 mM IPTG. Cells were harvested after 2 h of expression. All other preparation steps were done as described above^37^.

### Reverse labeling of the TEMPQANDSG and SHLYGWAFV samples

The expression protocol is nearly the same as above, with the following change: the pellet of the pre-culture was re-suspended in 1 L M9 minimal medium containing 50 mg of each of those amino acids (in ^15^N-labeled form) that should remain ^13^C-unlabeled, and 2 g of [1,3-^13^C]- or [2-^13^C]-glycerol and 0.5 g of [^15^N]-NH_4_Cl to label the sample name-giving amino acids with the desired pattern. All other preparation steps were done as described above^37^.

### Preparation of deuterated OmpG

^2^H, ^13^C, ^15^N-labeled OmpG was expressed on a fully deuterated M9 minimal medium containing [d_6_,^13^C]-glucose (2 g L^−1^ culture) and [d,^15^N]-NH_4_Cl (0.5 g L^−1^ culture) as sole carbon and nitrogen source, respectively. After purification under denaturing conditions (8 M urea), the proton content of the backbone amide groups was set to 70 or 100% by multiple buffer exchange. Both steps, refolding and reconstitution, were also performed in buffers containing either 70 or 100% H_2_O; the refolding buffer containing additionally 70 mM OG. 2D crystallization was achieved by dialysis using total or polar lipid extract from *E. coli* (yielding identical spectra) and a lipid to protein ratio of 1:2.

### Chemicals

Chemicals were purchased from the following suppliers: *n*-octyl-β-d-glycopyranoside (OG) and *n*-dodecyl-β-d-maltoside (DDM) from Glycon, Luckenwalde, Germany; *E. coli* total lipid extract or *E. coli* polar lipid extract from Avanti Polar Lipids, Alabaster, USA; Q-Sepharose Fast Flow and Resource-Q columns from GE Healthcare Europe, Freiburg, Germany. All other reagents were purchased from VWR International, Darmstadt, Germany, at the highest purity available.

### Proton-detected NMR

All proton-detected experiments were recorded on a narrow-bore 1000 MHz spectrometer equipped with a 1.3 mm triple-resonance MAS probe (Bruker, Karlsruhe, Germany). The MAS frequency was set to 60 kHz and the VT gas flow to 230 K, which roughly corresponds to a sample temperature of 300 K. Typical π/2-pulse lengths were 2.5 μs for ^1^H, 3.5 μs for ^13^C, and 5.5 μs for ^15^N. For the ^1^H/^15^N CP, a contact time of 700 μs was applied. A proton spin-lock with a 30% linear ramp centered on 8 kHz was used, whereas the ^15^N spins were locked with a square pulse with RF strength of 32 kHz. For the back transfer from ^15^N to ^1^H, a CP with duration of 300 μs was applied, with the proton spin-lock achieved by a 30% linear ramp centered on 5 kHz. The ^15^N spins were locked with a square pulse with RF strength of 34 kHz. Water suppression was achieved using the MISSISSIPI (multiple intense solvent suppression intended for sensitive spectroscopic investigation of protonated proteins, instantly) sequence without homospoil gradients^45^. Swept-low-power two-pulse phase modulation (TPPM) was used for ^1^H decoupling during nitrogen detection and WALTZ-16 for ^15^N and ^13^C decoupling during ^1^H-detection^46,47^. All spectra were acquired using States TPPI (time-proportional phase incrementation) in the direct dimensions to obtain pure phase line shapes and phase discrimination^48^. For the (H)NHH experiment, the effective acquisition time in the indirect dimensions was set to 4.7 and 12.1 ms for ^1^H and ^15^N, respectively. With eight scans per increment, the resulting total experiment time amounted 3 days. For the (H)N(HH)NH experiment, the acquisition time in the ^15^N dimension acquired before the through-space transfer was set to 15.4 ms. The acquisition time of the second ^15^N dimension, covering the ^15^N in the same amide group as the correlated ^1^H, was set to 10.7 ms. The number of scans per increment was 16 yielding a total experiment time of 7 days.

### Carbon-detected NMR

2D ^13^C-^13^C DARR spectra were recorded on a narrow-bore 900 MHz spectrometer equipped with a 3.2 mm triple-resonance MAS probe (Bruker, Karlsruhe, Germany). For all 2D experiments, the MAS frequency was set to 13 kHz and the sample temperature to 280 K. Typical π/2-pulse lengths were in the range 3.0–3.5 μs for ^1^H and around 5.0 μs for ^13^C. For the ^1^H/^13^C CP, a contact time of 1.5 ms was applied, using a proton spin-lock strength of 58.5 kHz (square pulse) and a carbon spin-lock strength ramped linearly around the *n* = 1 Hartmann–Hahn matching condition (50% ramp, optimized experimentally). During acquisition and indirect chemical shift evolution, a SPINAL64 (small phase incremental alternation with 64 steps) decoupling scheme with a RF strength of 90 kHz was applied to the proton spins. Various DARR mixing times, with durations of 20, 200, and 400 ms were used for the forward-labeled OmpG samples, whereas DARR mixing times of 50, 200, and 400 ms were used for reverse-labeled OmpG samples. The carrier frequency was placed at 100 ppm. Data were recorded and processed using Topspin version 2.1 (Bruker, Karlsruhe, Germany). The time domain data matrix of each experiment was 512 (*t*_1_) × 2048 (*t*_2_) points, with *t*_1_ and *t*_2_ increments of 10 and 16 μs, respectively. About 96 or 160 scans per point were recorded with a recycle delay of 3 s, resulting in total acquisition times of ~42 or 68 h, respectively. Data were processed with shifted-sinebell (in *t*_1_) and Lorentzian-to-Gaussian (in *t*_2_) apodization functions and zero filling was applied to 4096 (*t*_1_) × 8192 (*t*_2_) points. The carbon chemical shifts were indirectly referenced to 2,2-dimethyl-2-silapentane-5-sulfonic acid (DSS) by calibrating the downfield ^13^C adamantane signal to 40.48 ppm.

3D NCACX and NCOCX spectra were recorded on a wide-bore 400 MHz spectrometer equipped with a 3.2 mm triple-resonance MAS probe (Bruker, Karlsruhe, Germany). For all 3D experiments, the MAS frequency was set to 8 kHz and the sample temperature to 280 K. Typical π/2-pulse lengths were 3–3.5 μs for ^1^H, 5 μs for ^13^C, and 7 μs for ^15^N. For the ^1^H/^15^N CP, a contact time of 1.5 ms was applied, using a proton spin-lock strength of 55.0 kHz (square pulse) and a nitrogen spin-lock strength ramped linearly around the *n* = 1 Hartmann–Hahn matching condition (70% ramp, optimized experimentally). The ^15^N carrier frequency was set to 120 ppm. Following the evolution of nitrogen, adiabatic CP was employed to selectively transfer magnetization from ^15^N to either the Cα (NCA transfer) or the CO (NCO transfer). For the NCA-type experiments, the ^13^C carrier frequency was placed at 55 ppm and the RF spin-lock strengths were optimized to 3/2 *ω*_R_ for Cα and 5/2 *ω*_R_ for nitrogen, where *ω*_R_ is the MAS frequency, resulting to RF strengths of 12 and 20 kHz, respectively. For the NCO-type experiments, the ^13^C carrier frequency was placed at 170 ppm and the RF spin-lock strengths were optimized to 7/2 *ω*_R_ for CO and 5/2 *ω*_R_ for nitrogen, resulting to RF strengths of 28 and 20 kHz, respectively. For both NCA and NCO transfer, the ^15^N/^13^C CP contact time was optimized between 3 and 5 ms. For subsequent ^13^C homonuclear mixing, a DARR pulse sequence was used with various mixing times of 20, 50, 100, 200, and 400 ms, depending on the labeling scheme. During all acquisition and indirect chemical shift evolution periods, a SPINAL64 decoupling scheme was used with a RF strength of 90 kHz on the protons^49^. The 3D data sets were recorded using evolution times of 6.8 and 6.4 ms in *t*_1_ and *t*_2_, respectively. Each free induction decay was averaged from 96 scans, yielding a total measurement time of ~4 ½ days per spectrum.

### Torsion angle prediction for the structure calculations

The program TALOS+^22,23^ was used for prediction of torsion angles. Based on the chemical shift assignment, a reliable prediction was obtained for 128 φ and ψ torsion angles, yielding 256 torsion angle restraints in total.

### Distance restraints for the structure calculations

As input for the automated structure calculation using ARIA 2.3.2, lists with ambiguous distance restraints were produced by CCPN Analysis. The reason for using this rather than (unassigned) peak lists is that CCPN analysis supports the inclusion of complex isotope-labeling schemes as used in our studies into ARIA protocols. Still, the distance restraint lists were based on peak lists and produced using a CCPN macro script. This script is deposited in GitHub and can be downloaded under: https://github.com/jorenretel/ompg_restraint_generation. The script is detailed in the next two sections.

### ^1^H–^1^H distance restraints

ADRs were generated from (H)N(HH)NH and (H)NHH spectra as well as from 2D ^13^C–^13^C DARR spectra. For the (H)N(HH)NH and (H)NHH spectra, a 2.0 ms RFDR scheme was used for ^1^H homonuclear mixing. Chemical shift-matching of the peaks in these spectra to a dedicated chemical shift list (taking care of sample deuteration) was performed with a tolerance of 0.4 ppm in the ^15^N dimension(s) and 0.1 ppm in the indirectly detected ^1^H-dimension. For the directly detected ^1^H-dimension, a tolerance of 0.7 ppm was employed for shift-matching. Furthermore, the four-fold redundancy present in these spectra was used to decrease the amount of assignment possibilities for each restraint. This was done as follows: in cases that an assignment option for an ADR was supported by four peaks, other assignment options supported by only 1 or 2 peaks were removed. If the best assignment option present was supported by three peaks, assignment options only supported by one peak were removed. This yielded a set of 127 and 122 distance restraints for the (H)N(HH)NH and (H)NHH experiments, of which 42 and 41 distance restraints were unambiguous, respectively (Supplementary Table 2). The restraints were divided into two distance classes: 1.0–3.5 and 1.0–5.5 Å. This division was based on a simple sorting of the peak list by peak intensity. All peaks less or equally intense as the first peak for which a sequential assignment could be found (corresponding to a longer distance in the β-sheet) were classified in the distance class at 1.0–5.5 Å. All stronger peaks were classified in the distance class at 1.0–3.5 Å. These restraints were used as input to ARIA, which would further disambiguate those restraints that were left ambiguous.

### ^13^C–^13^C distance restraints

The ^13^C–^13^C distance restraints were obtained from a set of 11 spectra. The numbers of restraints are listed in Supplementary Table 2. The experiments can be divided into two groups, based on their mixing times. Medium mixing time (distance restraints in the class 1.5–5.5 Å): 2-OmpG, 200 ms DARR; 1,3-OmpG, 200 ms DARR; 2-TEMPQANDSG, 150 ms DARR; 1,3-TEMPQANDSG, 150 ms DARR, and 2-SHLYGWAFV, 150 ms DARR. Long mixing time (distance restraints in the class 1.5–7.0 Å): 2-OmpG, 400 ms DARR; 1,3-OmpG, 400 ms DARR; 2-TEMPQANDSG, 400 ms DARR; 1,3-TEMPQANDSG, 400 ms DARR; 2-SHLYGWAFV, 400 ms DARR; GAFY, 500 ms DARR. Peak picking was performed in the aliphatic region of the spectra. The ^13^C resonance assignment for this spectral region exceeds 90% with regard to the detected peaks, which is necessary for a successful structure calculation^50^. Furthermore, peaks were only picked in those regions of the spectra where no clusters of intra-residual signals were present. This was done to avoid generation of restraints from unassigned intra-residual peaks that can give rise to ADRs that do not contain a correct assignment option. Shift-matching was performed with a tolerance of 0.4 ppm in both ^13^C dimensions. The support of CCPN analysis for complex labeling schemes was exploited to pre-filter the assignment options for the ADRs, in a way that only those assignment options were kept that are consistent with the labeling scheme of the sample^51^. Only when the simultaneous labeling of the two carbon nuclei exceeded 10%, the assignment option was retained. ADRs were used as input to ARIA for further disambiguation. All ADRs based on the ^13^C-detected spectra were put into a single distance class with a lower bound of 1.5 Å and an upper bound of 8.0Å.

### Hydrogen bond restraints

No hydrogen bond restraints were added in the initial steps of the structure calculations since no experiments were performed to directly observe hydrogen bonds. However, after an initial structure was obtained (iteration 8 in Supplementary Fig. 13), the NMR restraint pattern corresponding to the hydrogen bonding observed in the β-sheet as a result of this run was inspected manually and hydrogen bond restraints were added in a subsequent full calculation that yielded the final structure in Supplementary Fig. 13. Accordingly, co-linear hydrogen bond restraints were created between every two residues for which the predicted dihedral angles indicated β-sheet and for which the full set of cross peaks appear in the ^1^H-detected spectra. Under these premises, 92 co-linear restraints (184 restraints in total) were produced using CCPN analysis^31,32^. The lower and upper bound for the H–O bond was set to the default values of 1.73 and 2.70 Å, respectively. For the N–O distances, these were 2.52 and 3.93 Å.

### Structure calculation protocol

The standard ARIA 2.3.2 protocol including the Ramachandran potential and CNS1.2 was used for structure calculations^52^. Both Cartesian and torsion angle dynamics were used. The refinement parameters as used in the simulated annealing procedure are displayed in Table 2. The applied default protocol consists of 9 iterations (numbered 0–8), followed by a refinement step in dimethyl sulfoxide (DMSO). Ensembles of 200 structures were calculated, starting from an extended backbone conformation. After each iteration, the 15 lowest-energy structures were selected from these ensembles for disambiguation of ADRs. This resulted in a modified list that was used in the subsequent round of structure calculation. ^1^H–^1^H distance restraints and torsion angle restraints entered the ARIA protocol in the first iteration. The more ambiguous ^13^C–^13^C restraints entered the protocol in iteration 4. A 4-to-4 restraint combination of the ^13^C–^13^C restraints was performed in the iterations 4–6. After that, standard merging of equivalent restraints was performed. Hydrogen bond restraints were used in the final structure calculation run.

**Table 2:** Refinement parameters used in the simulated annealing procedure

TAD high temperature	20,000 K
TAD time step factor	9.0
Cartesian high temperature	3000 K
Time step	0.003
Final temperature cool stage 1	1000 K
Steps in cool stage 1	100,000
Final temperature cool stage 2	50 K
Steps in cool stage 2	100,000
High-temperature steps	20,000
Refine steps	8000

### Data availability

All relevant data necessary for producing the samples, assigning the protein signals, and calculating the structures are available from the corresponding author upon reasonable request. The NMR data and protein structure are deposited in the BioMagResBank (BMRB) with ID 34088 and the Protein Data Bank (PDB) with ID 5MWV, respectively. The script is deposited in GitHub and can be downloaded under: https://github.com/jorenretel/ompg_restraint_generation.

## Electronic supplementary material

Supplementary Information
