# Supplementary material for: Structure of outer membrane protein G in lipid bilayers
Source: Nat Commun. 2017 Dec 12;8:2073. doi: 10.1038/s41467-017-02228-2 (PMC5727033; doi:10.1038/s41467-017-02228-2)
Supplement: Supplementary file 1 — Supplementary Information [file 41467_2017_2228_MOESM1_ESM.pdf]

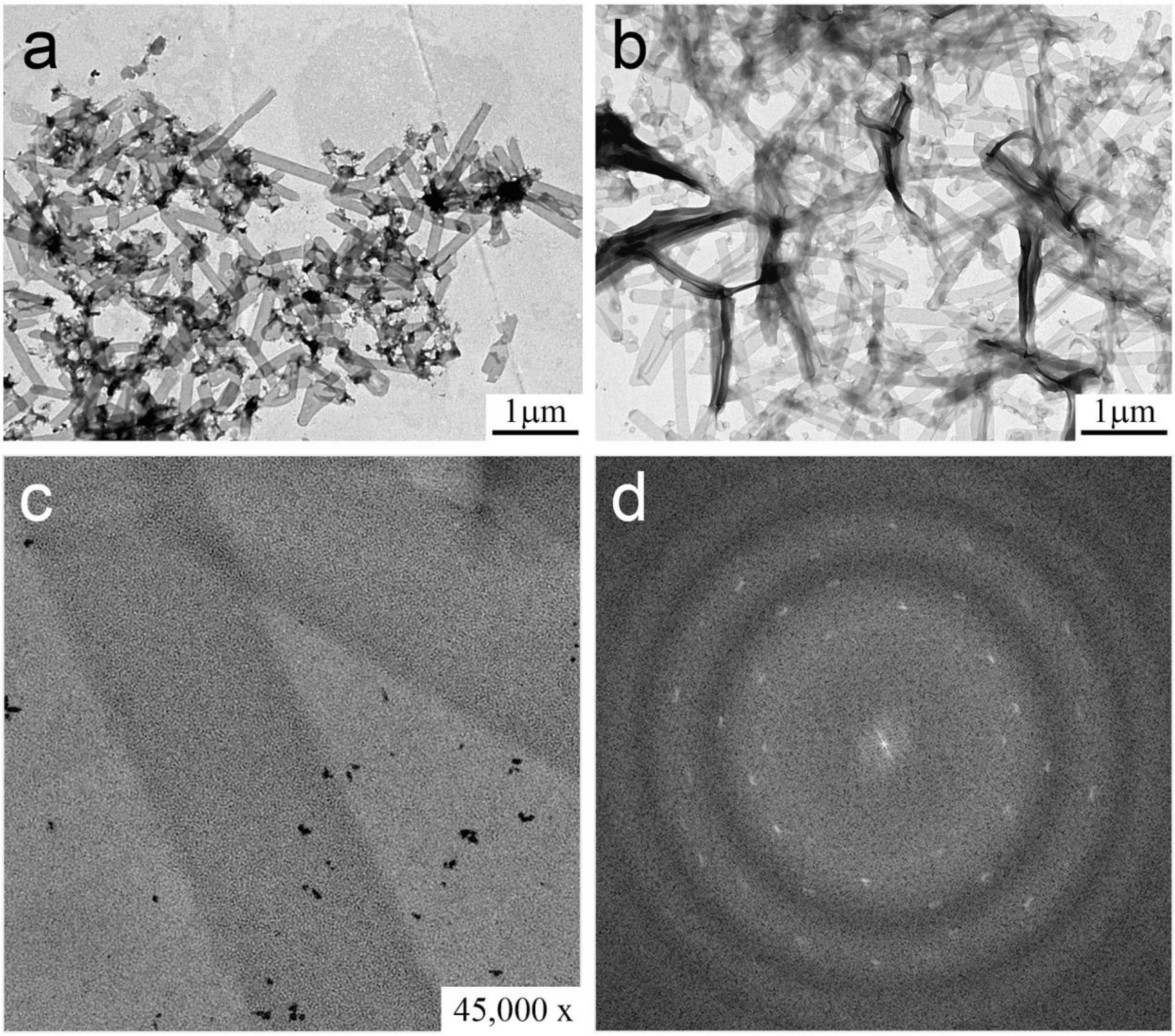

Supplementary Figure 1. Electron microscopy and diffraction data of OmpG

Electron microscopy of different isotopically labelled OmpG preparations after reconstitution into *E. coli* lipids and growth of 2D crystals at low magnification showing long tubes with a width of 200-300 nm. a) 2D crystals of the 1,3-OmpG [TEMPQANDSG] sample (see Fig. 1c of the main text), negative stain; b) 2D crystals of the 2-OmpG [SHLYGWAFV] sample (see Fig. 1d), negative stain; c) High magnification image of a negative stained 2D crystal of uniformly ^{13}C,^{15}N-labelled OmpG; d) diffraction pattern of the sample shown in c.

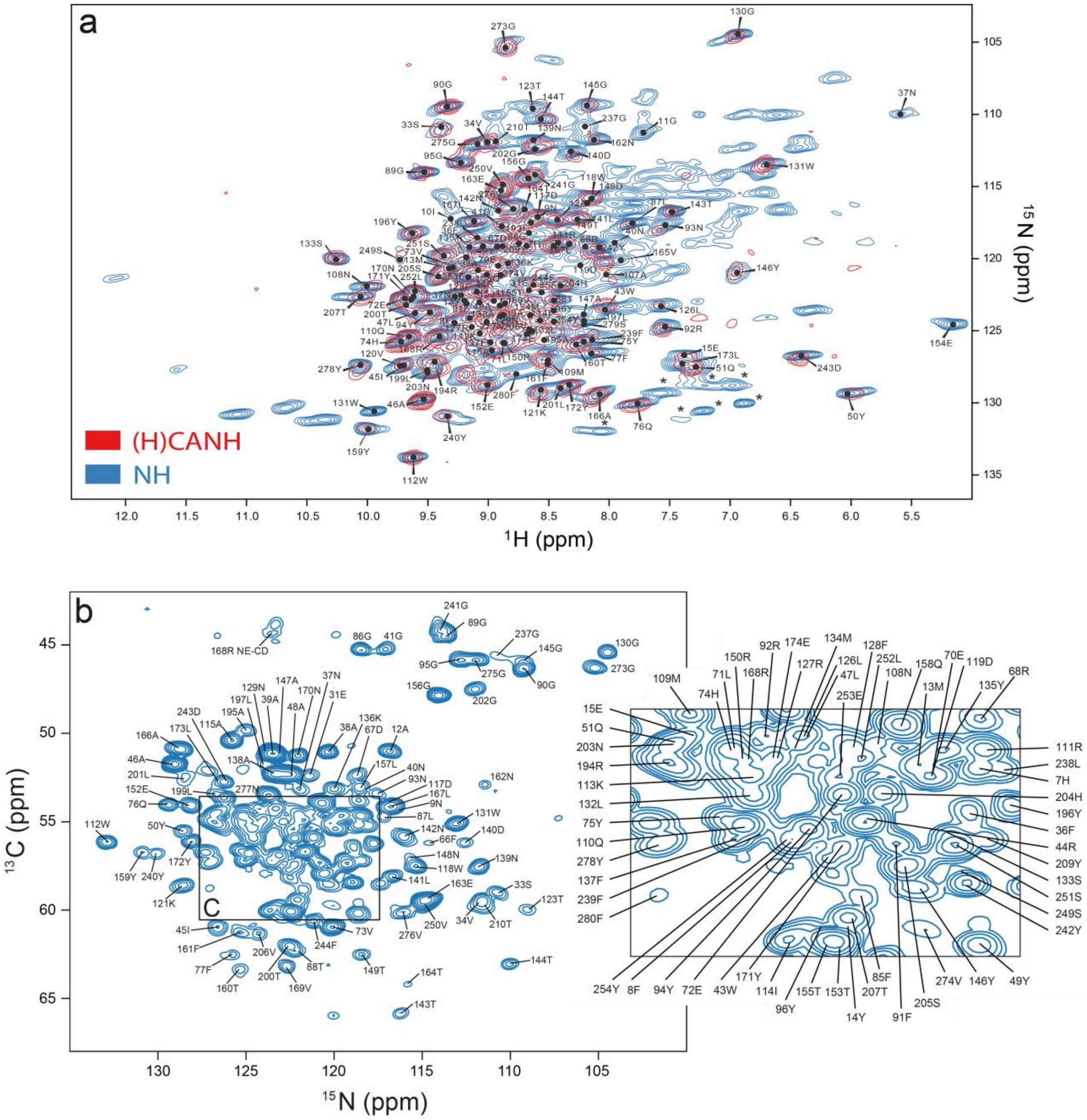

Supplementary Figure 2. Sequence-specific assignment of OmpG

a) Superposition of a CP-based ^{15}N-^{1}H correlation (blue) of OmpG in lipid bilayers and a projection of the (H)CANH spectrum (red). Asterisks indicate folded-in arginine side-chain signals. Their position in the two spectra is different since the respective spectral width in the ^{15}N-dimension was set differently. b) ^{13}C-^{15}N-projection of the (H)CANH spectrum with assignments. The dispersion in this projection is larger than in the ^{15}N-^{1}H projection. The majority of peaks is assigned (151). 31 peaks of the (H)CANH spectrum remain unassigned.

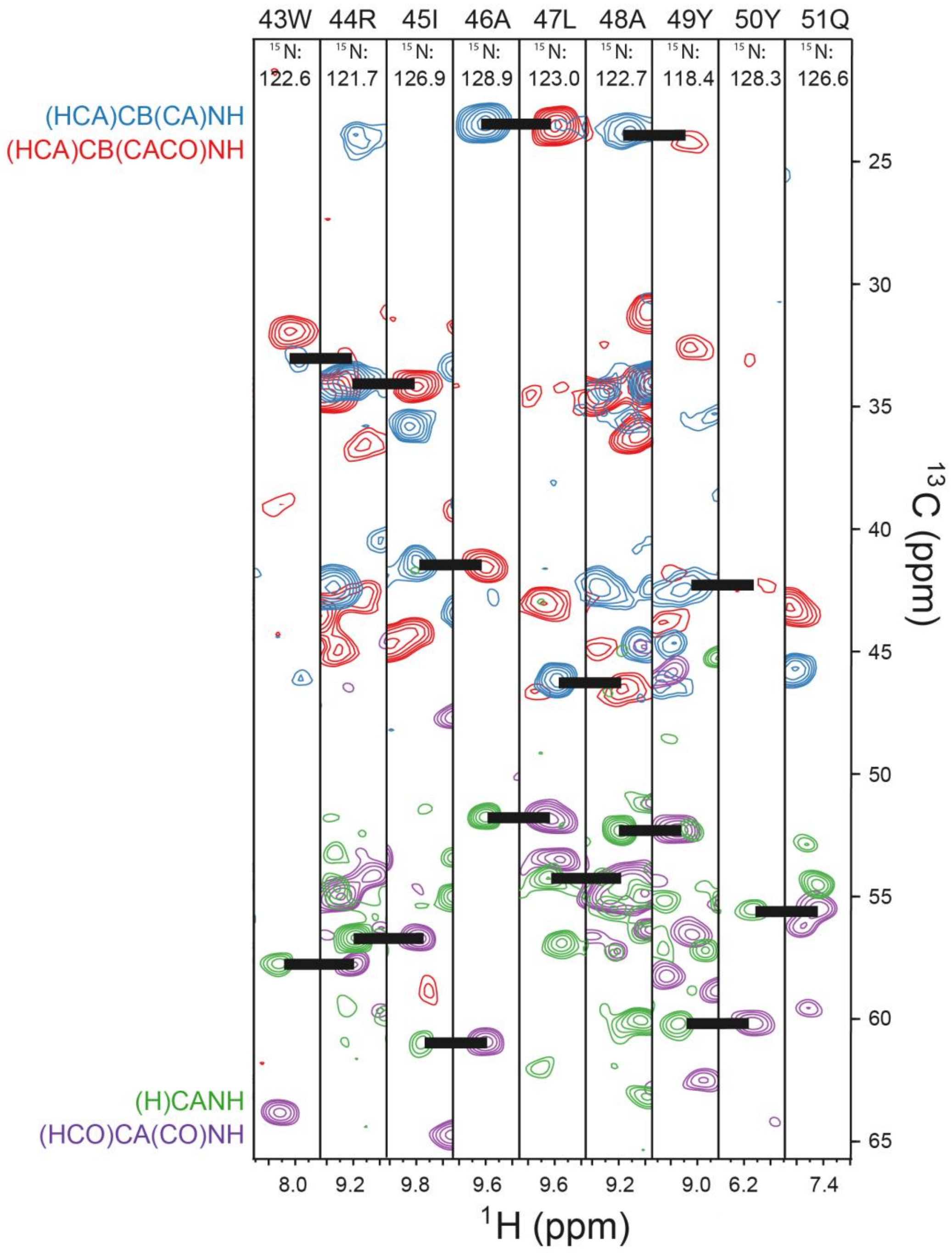

Supplementary Figure 3. Strips from 3D NMR correlation spectra of OmpG to illustrate sequence-specific assignment

Superposition of strip plots taken from the (H)CANH, (HCO)CA(CO)NH, (HCA)CB(CA)NH and (HCA)CB(CACO)NH spectra at the ^{15}N-chemical shifts of the residues in the subsequence tryptophan 43 to glutamine 51. Residue 42 is a proline, and cross peaks correlating its Cα and Cβ are present in the tryptophan 43 strip of the (HCO)CA(CO)NH and (HCA)CB(CACO)NH spectra. The signal intensities of the peaks in the experiments that contain multiple polarization transfer steps [(HCA)CB(CA)NH and (HCA)CB(CACO)NH] drop off towards the end of this sequential stretch of amino acids and eventually completely disappear in the strip of glutamic acid 51, which is the last assigned residue of this β-strand.

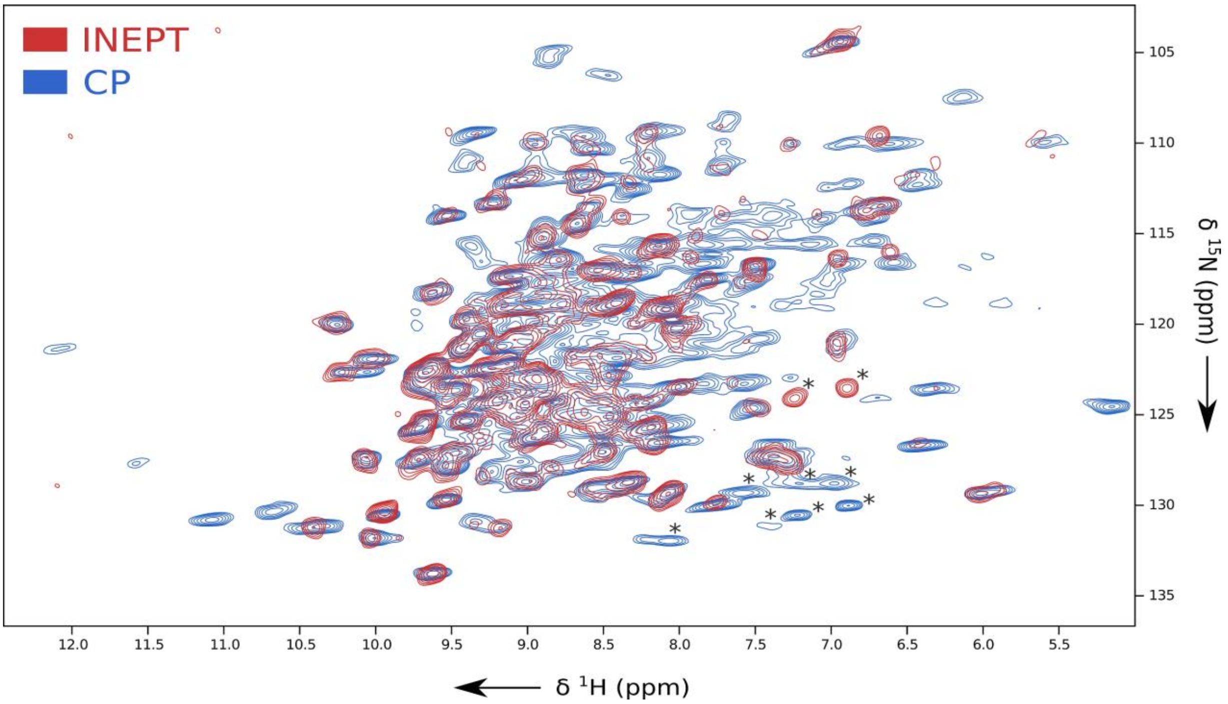

Supplementary Figure 4. Comparison of CP-based and INEPT-based NMR experiments

Superposition of a CP-based ^{15}N-^{1}H correlation (blue) of OmpG in lipid bilayers and an INEPT-based ^{15}N-^{1}H correlation (red). Asterisks indicate folded-in arginine side-chain peaks. Their position in the two spectra is different since the respective spectral with in the ^{15}N-dimension was set differently.

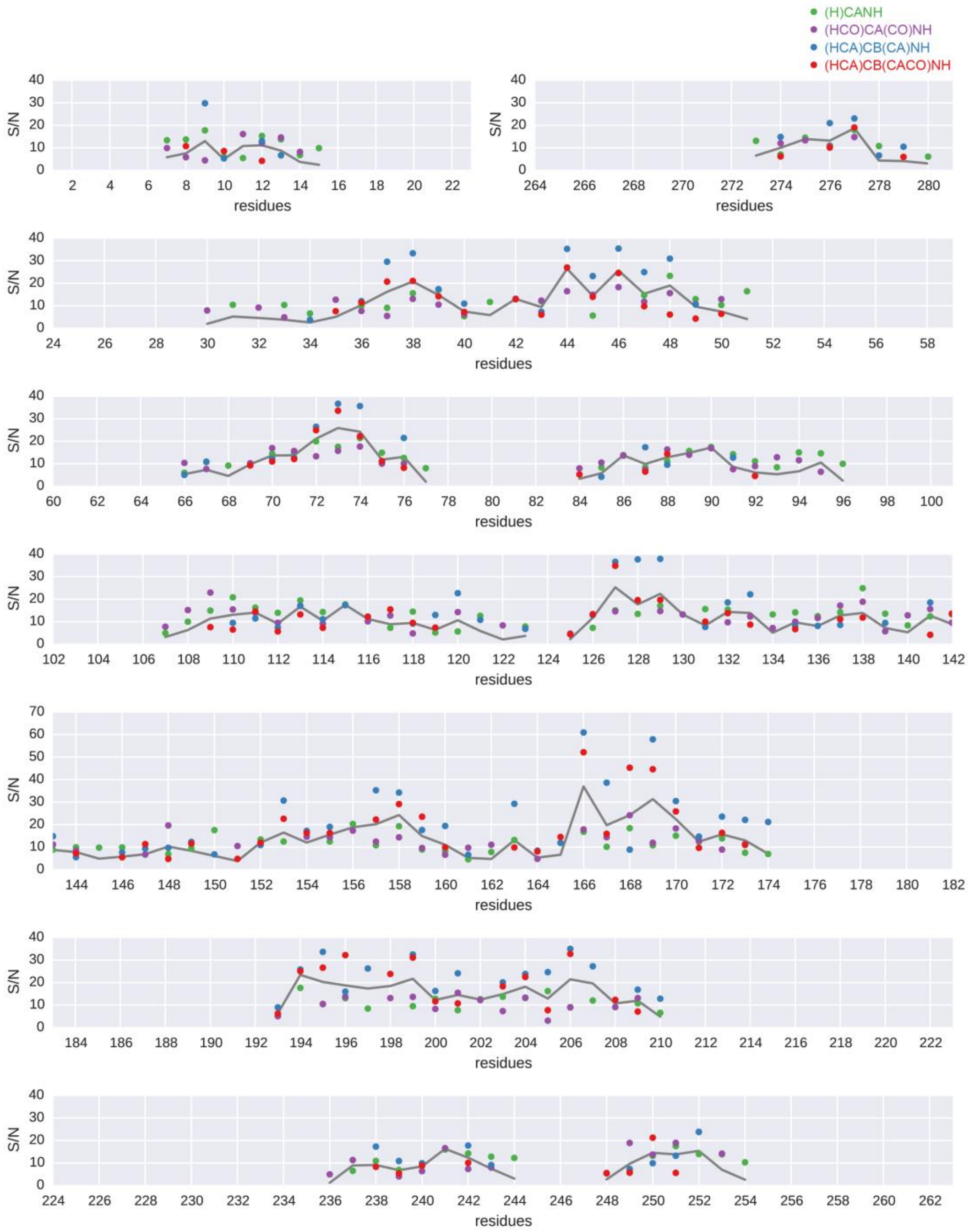

Supplementary Figure 5. Comparison of cross-peak intensities in different proton-detected 3D NMR experiments

Signal intensities in the (H)CANH, (HCO)CA(CO)NH, (HCA)CB(CA)NH and (HCA)CB(CACO)NH spectra, plotted as signal-to-noise versus sequence. Every panel represents two strands in the β-sheet connected by an intracellular turn in the structure, except for the two panels on the top, which represent the first and last strand of the sequence. For all peaks, residue indices are based on the location of the excited carbon. Thus, peak intensities in the (HCO)CA(CO)NH and (HCA)CB(CACO)NH spectra correspond to strips at the ^{15}N-^{1}H position of the residue with index +1. The noise level is defined as one standard deviation of noise intensity calculated within CCPN analysis by taking 10 subsets of 1000 random samples in a spectrum and choosing the smallest subset. The solid line represents a S/N average.

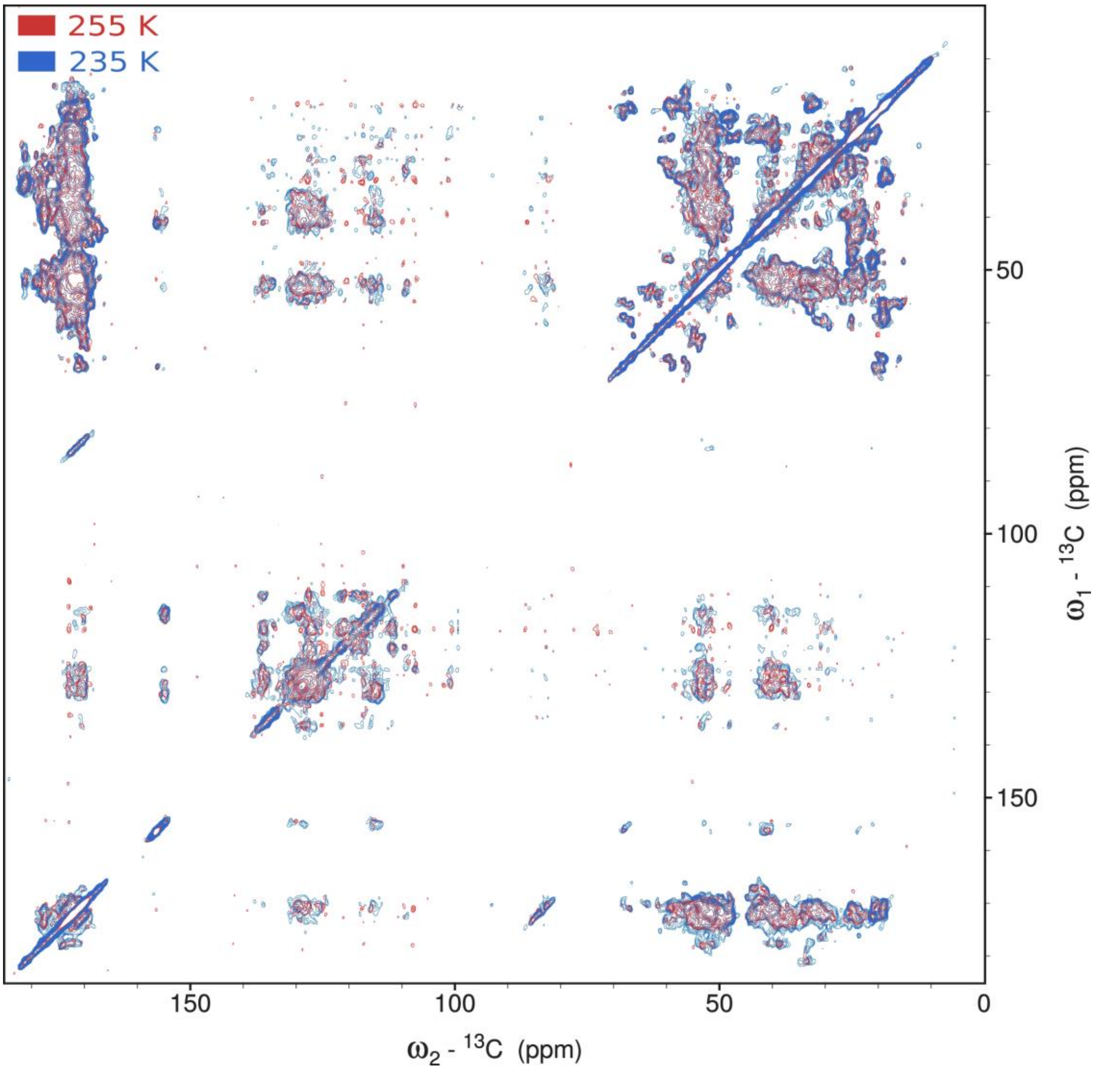

Supplementary Figure 6. Comparison of cross peak patterns at different temperatures

In order to check whether additional signal sets or increased signal intensity could be observed at lower temperatures, ^{13}C-^{13}C correlation spectra with 50 ms DARR mixing were recorded at 255 K (red) and 235 K (blue). The temperature in the latter spectrum is underneath the lipid phase-transition as monitored by a change in the 1D ^{1}H spectrum. No significant difference between the two ^{13}C-^{13}C spectra was observed.

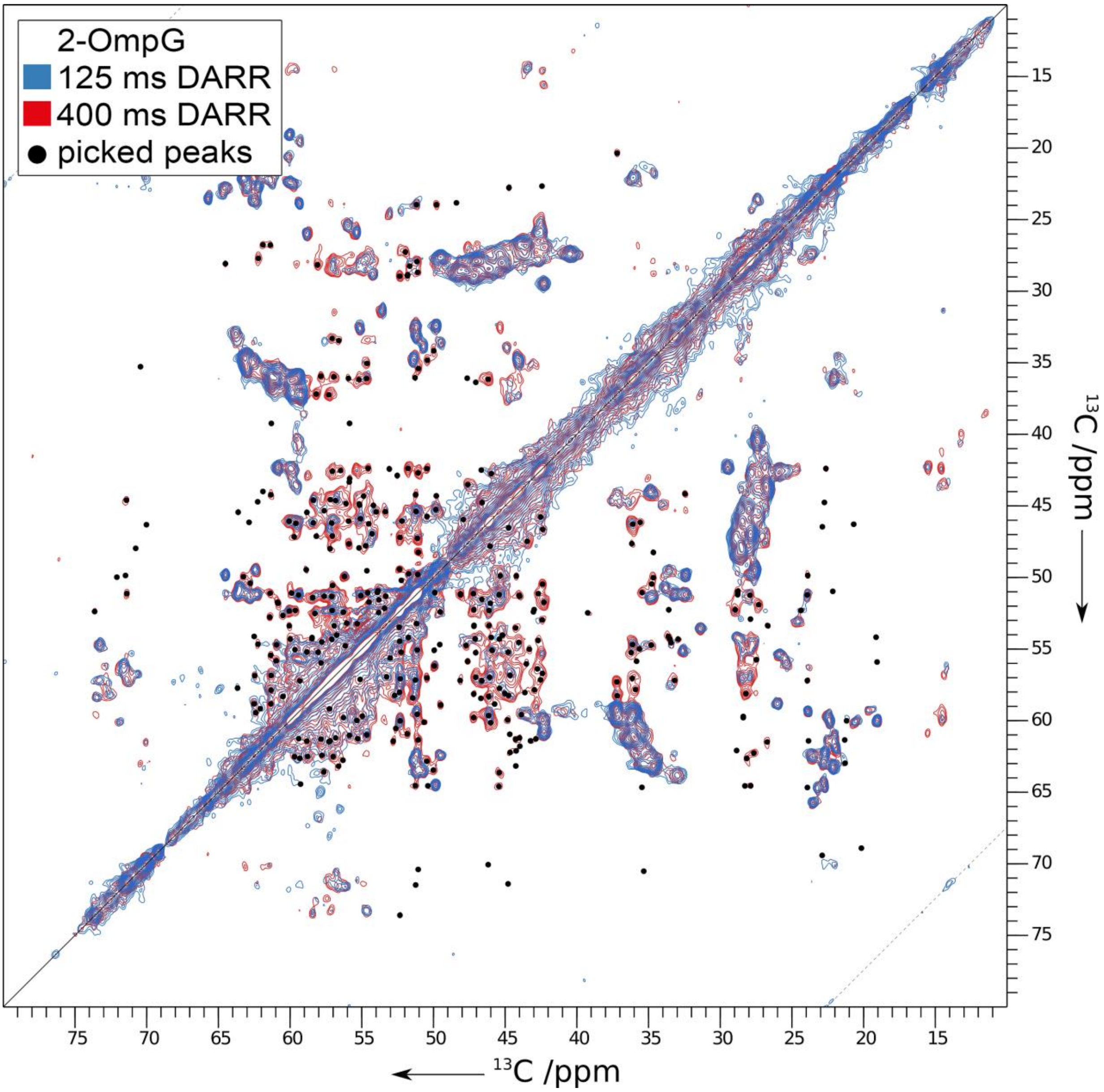

Supplementary Figure 7. Selection of inter-residual cross peaks for distance restraints for the 2-OmpG sample

Peaks used to generate distance restraints in the ^{13}C-^{13}C correlation (400 ms DARR) of the 2-OmpG sample. Intra-residual peaks were avoided during peak picking. This was helpful because some intra-residual peaks can correspond to unassigned spin systems. If these peaks would be included in the shift matching procedure, restraints lacking a correct assignment option would be produced, which is highly unfavorable. To avoid such intra-residual peaks, the spectrum was compared with a spectrum recorded with a shorter mixing time. Furthermore, knowledge of the specific areas in which intra-residual peaks can be expected is used. For instance, within the C_α-C_α area close to the diagonal (50-60 ppm) no intra-residual are expected except for proline C_α-C_δ correlations and serine outliers, hence also peaks present in both short and long mixing time spectra were picked (except for the respective proline C_α-C_δ peaks).

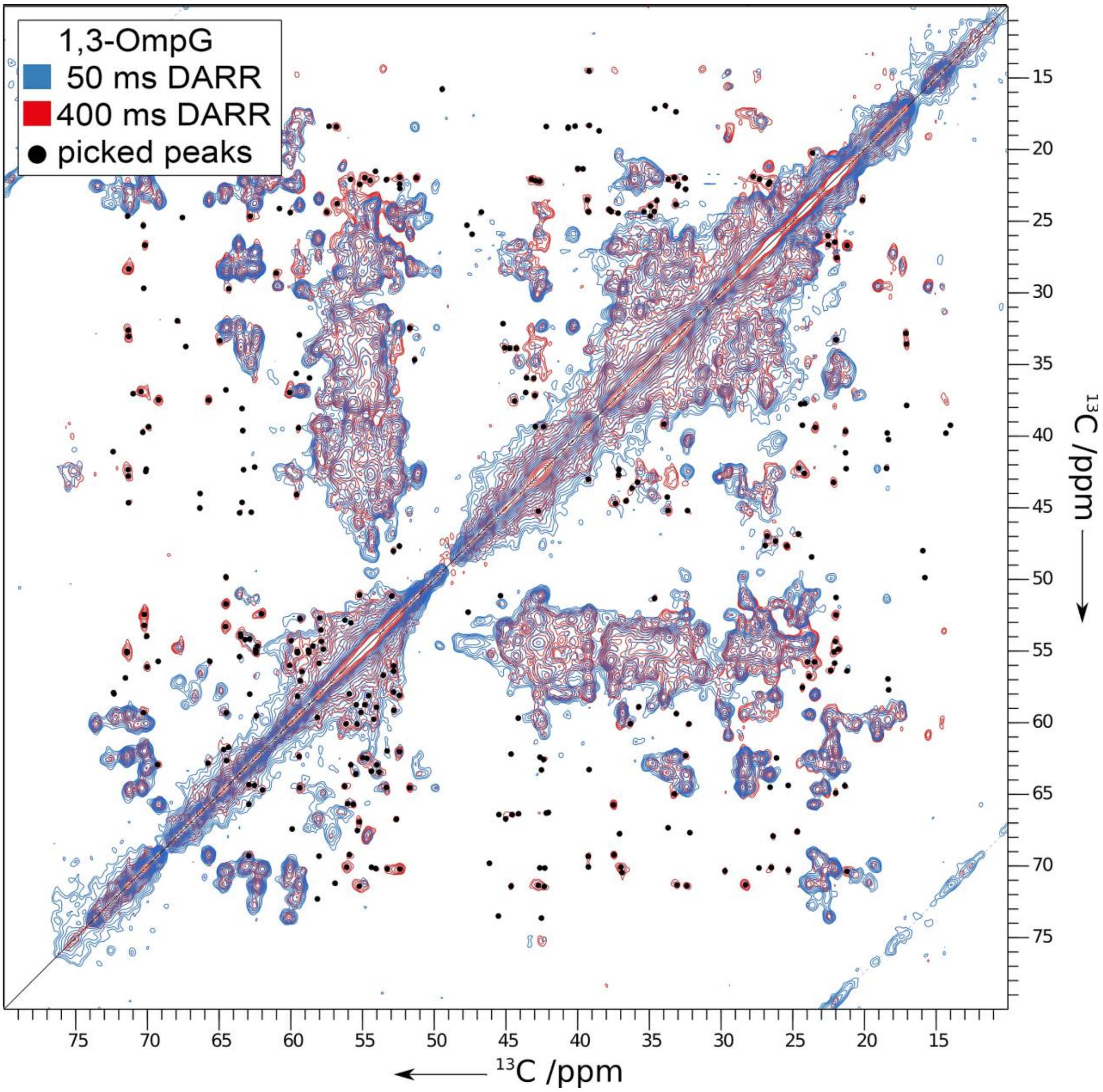

Supplementary Figure 8. Selection of inter-residual cross peaks for distance restraints for the 1,3-OmpG sample

Peaks used to generate distance restraints in the ^{13}C-^{13}C correlation spectrum (400 ms DARR mixing) of the 1,3-OmpG sample. See also legend of Supplementary Figure 7.

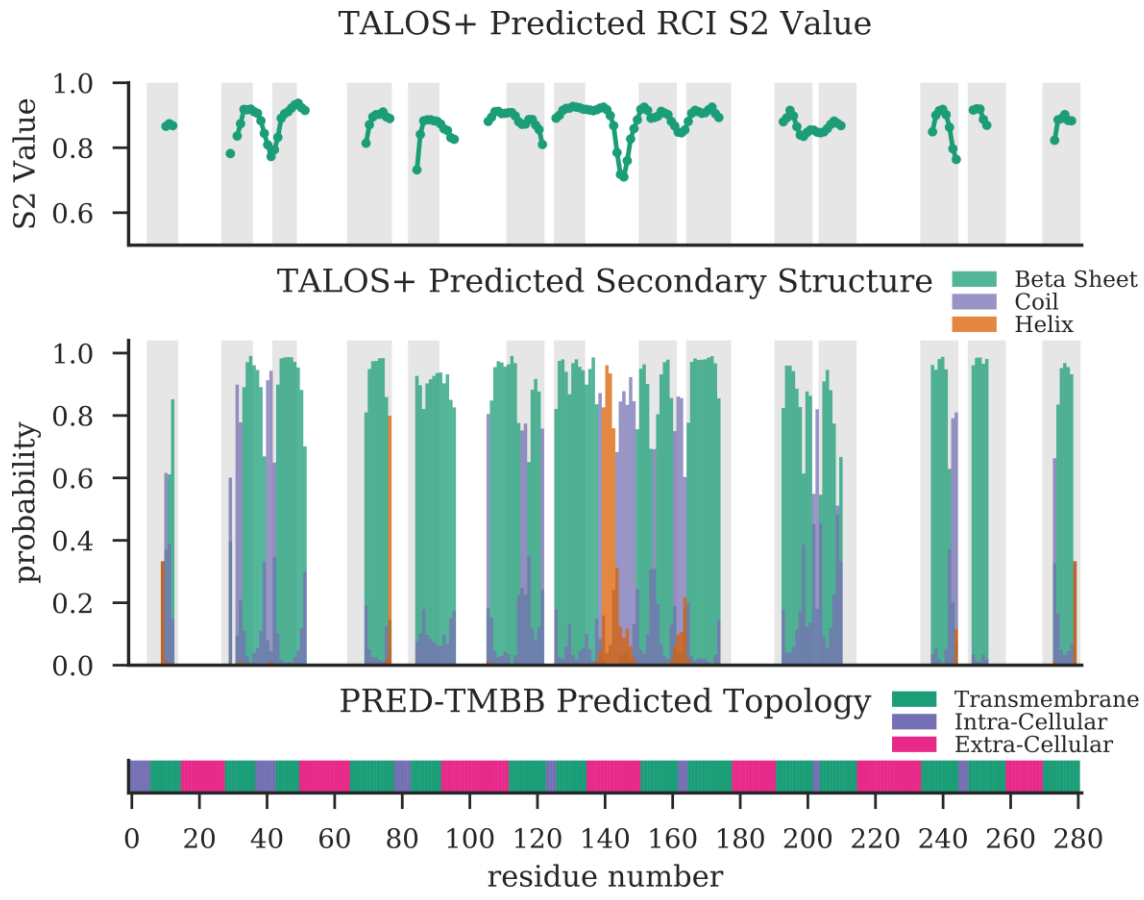

Supplementary Figure 9. Secondary structure prediction

Prediction of secondary structure based on chemical shifts by TALOS+[1,2] and PRED-TMBB[3]. TALOS+ uses the secondary chemical shifts of assigned residues to search a database for triplets in the sequence of high-resolution structures with similar secondary chemical shifts to predict φ/ψ torsion angles. PRED-TMBB is an algorithm that solely relies on the sequence and predicts which parts of the sequence are intra-cellular, extra-cellular and transmembrane, given the molecule is a transmembrane β-barrel. Grey areas in the TALOS+ plot correspond to those regions of the sequence that are predicted as transmembrane by PRED-TMBB.

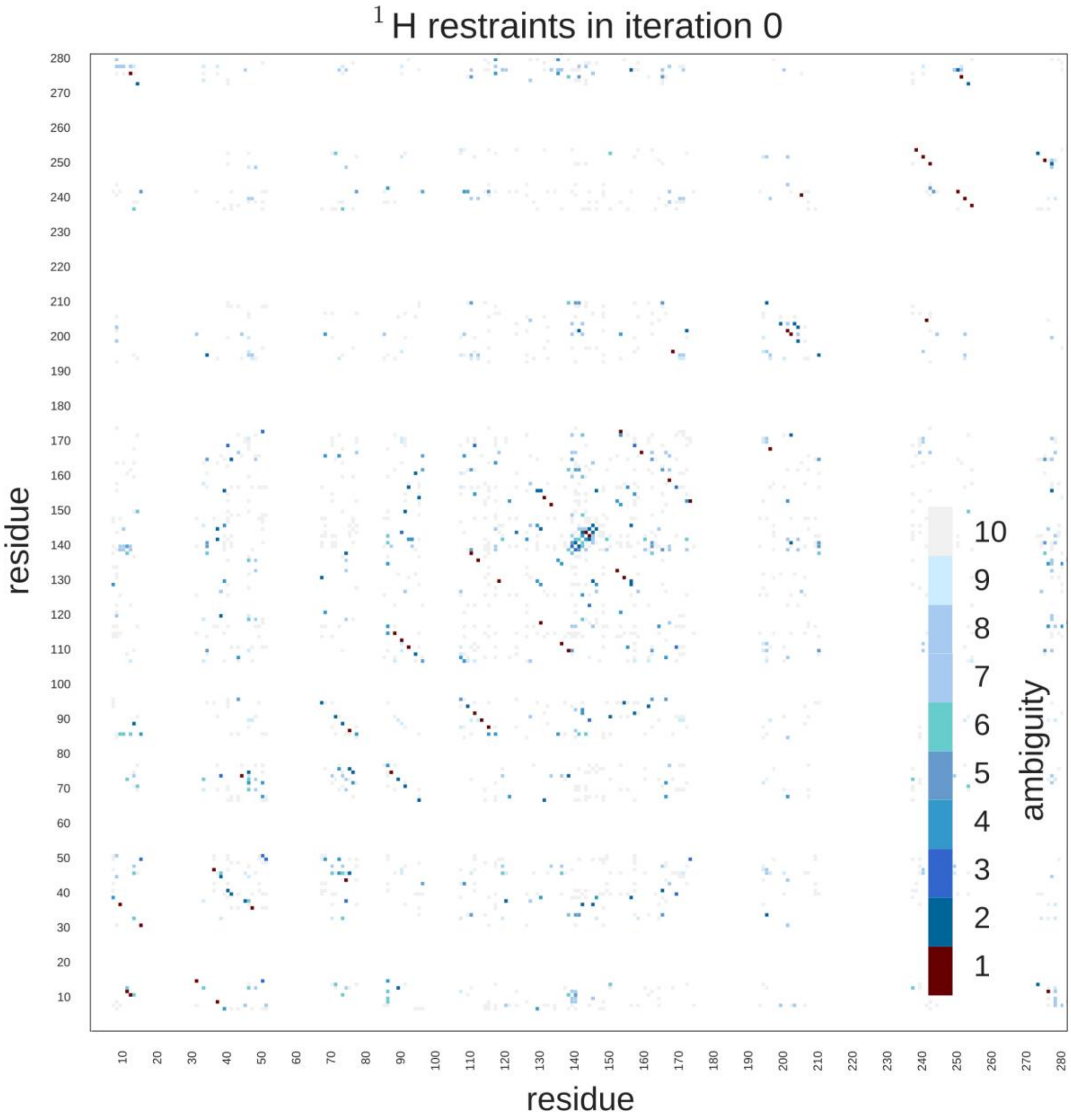

Supplementary Figure 10. **Interaction matrix of ^{1}H-^{1}H distance restraints before disambiguation**

Residue interaction matrix for ^{1}H-^{1}H distance restraints entering the ARIA protocol (before any disambiguation by ARIA). The color indicates the ambiguity of the least ambiguous restraint present for the interaction between two residues. Interactions between two residues for which an unambiguous restraint is present are colored red. Patterns perpendicular to the diagonal, indicating anti-parallel β-sheets, can already be observed.

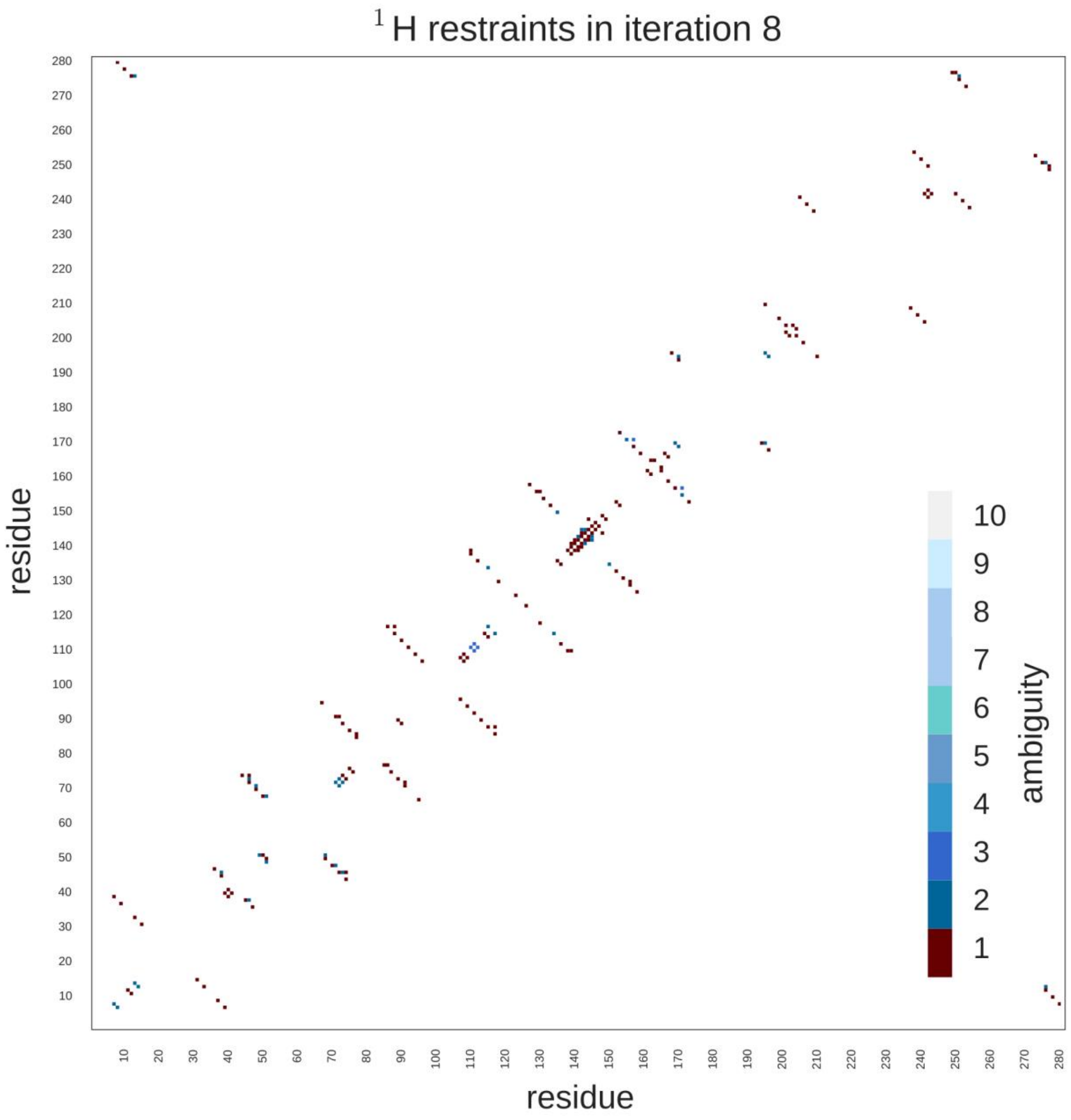

Supplementary Figure 11. Assignment status of ^{1}H-^{1}H ADRs after the last iteration of the ARIA protocol

The color indicates the ambiguity of the least ambiguous restraint present for the interaction between two residues. Interactions between two residues for which an unambiguous restraint is present are colored red. A clear alternating pattern can be seen for the β-sheets perpendicular to the diagonal. 11 restraints in the ^{1}H-^{1}H restraint set were left ambiguous at the end of the ARIA procedure.

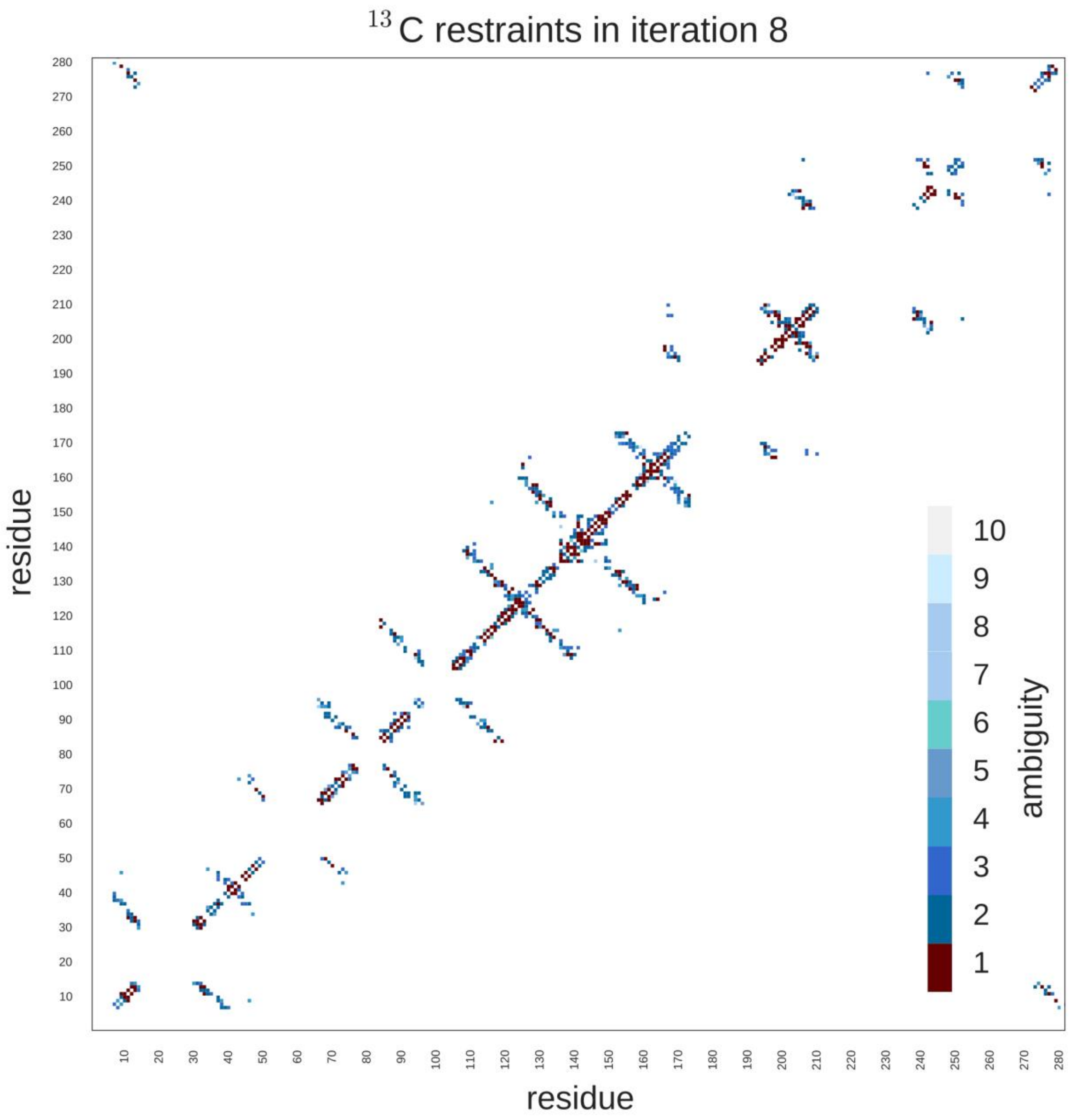

Supplementary Figure 12. Assignment of ^{13}C-^{13}C ambiguous distance restraints in the last iteration of the ARIA protocol

The color indicates the ambiguity of the least ambiguous restraint present for the interaction between two residues. Interactions between two residues for which an unambiguous restraint is present are colored red. 488 restraints in the ^{13}C-^{13}C restraint set were left ambiguous at the end of the ARIA procedure.

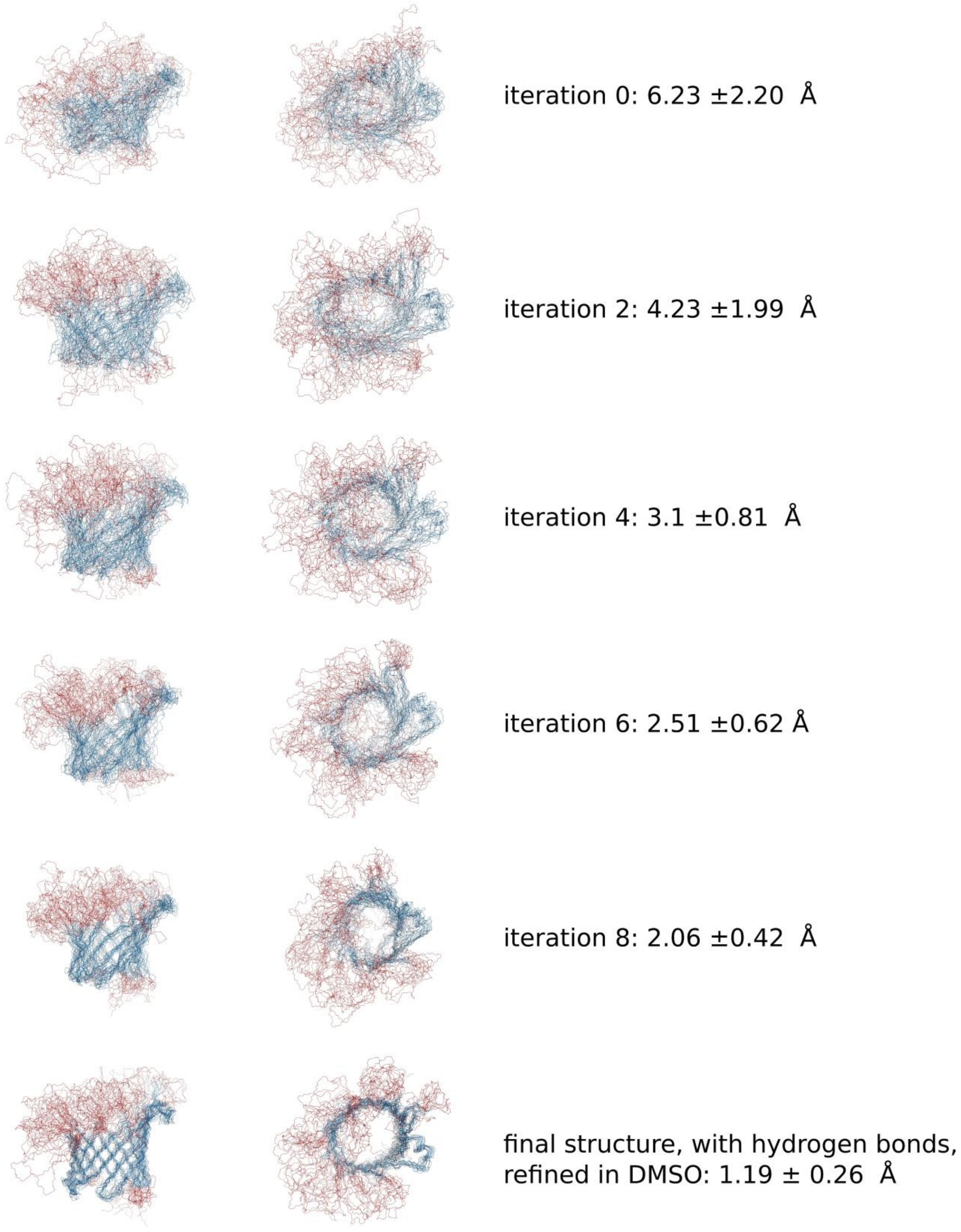

Supplementary Figure 13. Ensembles of the 15 lowest energy structures

The 15 lowest energy structures out of 200 calculated structures are shown (from top to bottom) after iterations 0, 2, 4, 6 and 8 of the ARIA protocol, and the final structure refined with explicit hydrogen bond restraints (bottom).

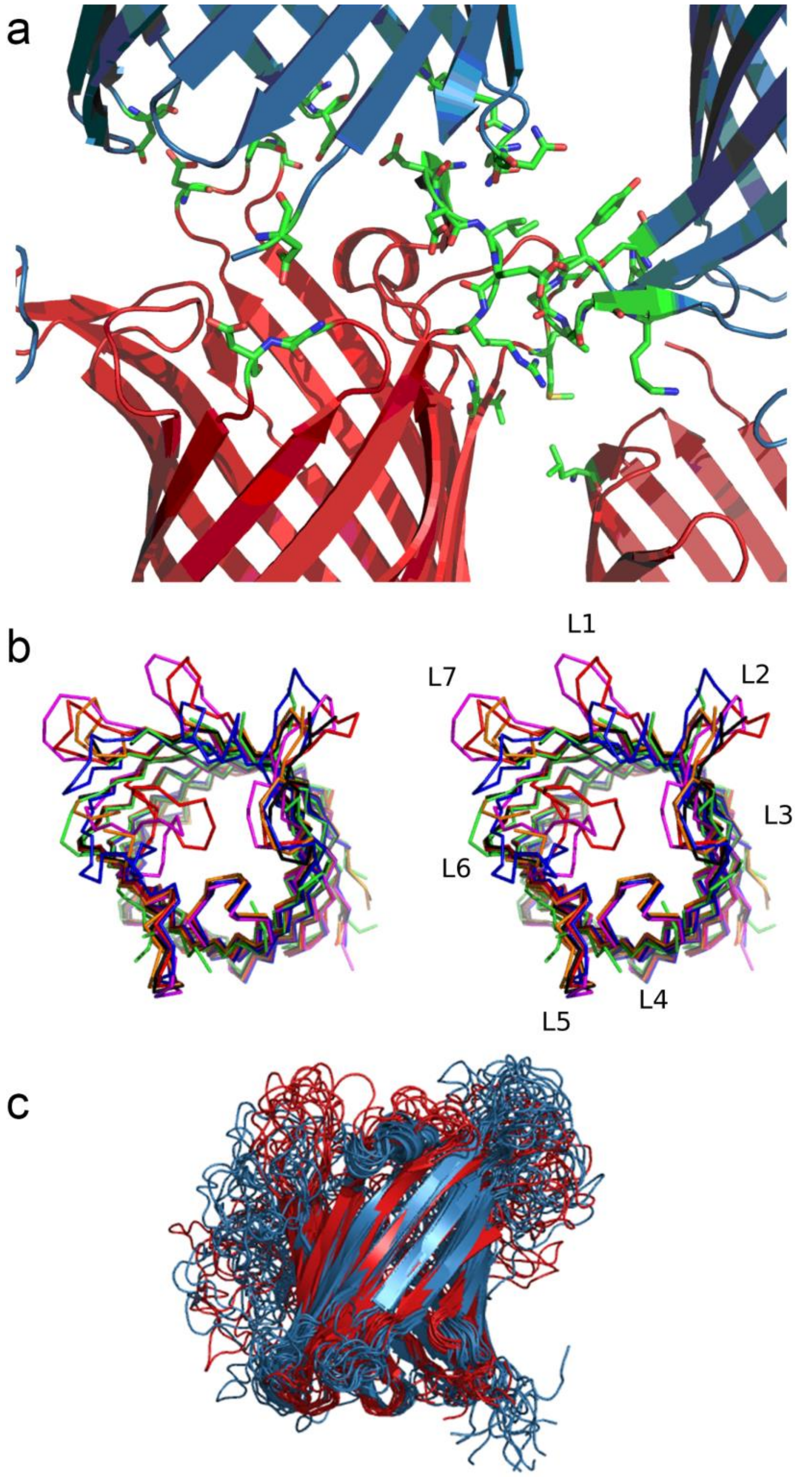

Supplementary Figure 14. Comparison of the solid-state NMR-based structure with X-ray- and solution NMR-based structures

a) Crystal contacts between four protein molecules in the X-ray structure 2IWV[4]. b) Superposition of six different X-ray structures of OmpG: green: 4CTD[5], red: 2IWW[4], blue: 2IWV[4], black: 2F1C[6], magenta: 2X9K[7], orange: 2WV[8]. c) Overlay of solid-state NMR (blue) and liquid-state NMR (red) structural ensembles.

	assigned nuclei	% within 170 assigned residues	% of whole sequence
**^{13}C-detected**			
N backbone	124	73% (124/170)	44% (124/281)
C'	117	69% (117/170)	42% (117/281)
CA	164	96% (164/170)	58% (164/281)
CB	146	94% (146/156)	57% (146/254)
C aliphatic	449	93% (449/485)	57% (449/781)
C aromatic	58	26% (58/227)	17% (58/341)
C carbonyl	127	62% (127/204)	35% (127/360)
**^{1}H-detected**			
H backbone	151	92% (151/164)	56% (151/272)
N backbone	151	89% (151/170)	54% (151/281)
C'	131	77% (131/170)	47% (131/281)
CA	167	98% (167/170)	59% (167/281)
CB	131	84% (131/156)	52% (131/254)

Supplementary Table 1. Comparing the extend of chemical assignment on basis of ^{13}C-detected and ^{1}H-detected experiments

Extend of the chemical shift assignment achieved for different atom sites with regards to signals observed in ^{13}C-detected experiments (on fully protonated samples) and ^{1}H-detected experiments (on deuterated and back-exchanged samples). The numbers of assigned chemical shifts are listed separately for the two types of samples/measurement methods because isotope shift causes the chemical shifts to be slightly different for these two types of samples. Therefore, chemical shift assignment for one type of sample does not automatically lead to the assignment in the other type of sample, and in few individual cases it was difficult to transfer the assignment. The extent of the assignment is given as a percentage of the total number of nuclei (of the respective kind) in the whole protein and as a percentage of the number of nuclei within the 170 residues for which at least one nucleus is assigned (colored blue in Fig. 1a of the main text).

distance restraints	all	^{1}H-detected	(H)NHH	(H)N(HH)NH	^{13}C-detected	2-OmpG 400 ms	2-OmpG 200 ms	1,3-OmpG 400 ms	1,3-OmpG 200 ms	2-TEMPQANDSG 400 ms	2-TEMPQANDSG 150 ms	1,3-TEMPQANDSG 400 ms	1,3-TEMPQANDSG 150 ms	2-SHLYGWAFV 400 ms	2-SHLYGWAFV 150 ms	GAFY 500 ms
**input to ARIA**																
total	**2096**	249	122	127	1847	355	196	312	146	131	60	234	32	216	84	81
unambiguous	**103**	83	41	42	20	0	0	0	0	4	1	10	0	4	0	1
rejected during protocol	**249**	8	5	3	241	14	17	34	14	18	10	51	3	51	16	13
**assignment in ARIA iteration 8**																
total	**1847**	241	117	124	1606	341	179	278	132	113	50	183	29	165	68	68
distance class < 3.5 Å	**139**	139	66	73												
distance class < 5.5 Å	**560**	102	51	51	458		179		132		50		29		68	
distance class < 8.0 Å	**1148**				1148	341		278		113		183		165		68
intra-residual	**31**	0	0	0	31	14	4	8	1	0	0	4	0	0	0	0
sequential	**1197**	64	37	27	1133	236	140	193	99	79	37	131	27	94	51	46
medium-range (2 ≤|i-j|<5)	**184**	13	6	7	171	34	11	42	14	5	0	20	0	29	6	10
long-range (|i-j|≥ 5)	**435**	164	74	90	271	57	24	35	18	29	13	28	2	42	11	12
unambiguous	**1071**	222	105	117	849	142	62	131	68	95	41	131	19	88	34	38
unique	**880**	109	78	79	771	239	132	193	87	71	33	122	19	119	43	49
uique long-range	**215**	57	44	53	158	41	20	23	11	21	9	22	2	33	7	12
unique unambiguous long-range	**132**	53	42	51	79	8	6	11	5	15	7	17	2	23	5	5
**violations in dmso refined OmpG**[a]																
> 0.3 Å	**1**	0	0	0	0	0	1	0	0	0	0	0	0	0	0	0
> 0.5 Å	**0**	0	0	0	0	0	0	0	0	0	0	0	0	0	0	0

torsion angle restraints	
φ/ψ angles	128 (256 total)
rmsd	0.880 ± 0.098
**violation count per model**	
violations > 1°	24.9 ±3.8
violations > 3°	5.8 ±2.0
violations > 5°	1.5 ±0.7
violations > 10°	0

hydrogen bond restraints	
92 colinear restraints (184 total)	
violation > 0.3 Å	0

backbone rmsd[b]	
β-sheet residues	1.19 ±0.26 Å
vs. 2IWW (x-ray)	1.94 ±0.17 Å
vs. 2IWV (x-ray)	1.93 ±0.19 Å
vs. 2F1C (x-ray)	1.96 ±0.19 Å
vs. 2JQY (solution NMR)	2.03 ±0.21 Å
β-sheet + turn residues	1.49 ±0.40 Å
all residues	5.29 ±0.54 Å

PROCHECK	
ramachandran	
core:	93.7%
allowed:	5.5%
generous:	0.4%
disallowed:	0.5%

WHATIF	
Structure Z-scores, positive is better than average:	
1st generation packing quality	0.356 ±1.201
2nd generation packing quality	1.706 ±1.403
Ramachandran plot appearance	0.499 ±0.246
chi-1/chi-2 rotamer normality	3.047 ±0.529
Backbone conformation	0.547 ±0.206
RMS Z-scores, should be close to 1.0:	
Bond lengths	0.971 ±0.001
Bond angles	0.318 ±0.003 (tight)
Omega angle restraints	0.723 ±0.035 (tight)
Side chain planarity	0.327 ±0.026 (tight)
Improper dihedral distribution	0.398 ±0.007
Inside/Outside distribution	1.214 ±0.015 (unusual)

Supplementary Table 2. Statistics on the restraints and quality metrics of the 15 lowest energy structures

All quality measures correspond to the structure refined in DMSO. Structure validation was performed using the iCing server[9] from which PROCHECK[10] and WHATIF[11] were obtained. Precise counts for specific restraint subsets were obtained using a CCPNMR Analysis macro. a) Numbers span the complete ensemble. One distance restraint violation was present in 1 of the 15 models. b) Alignment of models within the ensemble and with structures 2IWW and 2IWV[4], 2F1C[6] and 2JQY[12] were calculated using biopython[13]. The β-sheet extends over residues 7-15, 33-40, 43-50, 69-77, 84-94, 109-121, 126-138, 150-160, 166-174, 193-201, 204-210, 237-243, 248-254 and 273-279. Turn residues are 41-42, 78-83, 122-125, 161-165, 202-203 and 244-247.

Supplementary References

1. Cornilescu G, Delaglio F, Bax A. Protein backbone angle restraints from searching a database for chemical shift and sequence homology. *J Biomol NMR* **13**, 289-302 (1999).
2. Shen Y, Delaglio F, Cornilescu G, Bax A. TALOS plus : a hybrid method for predicting protein backbone torsion angles from NMR chemical shifts. *Journal of Biomolecular Nmr* **44**, 213-223 (2009).
3. Bagos PG, Liakopoulos TD, Spyropoulos IC, Hamodrakas SJ. PRED-TMBB: a web server for predicting the topology of beta-barrel outer membrane proteins. *Nucleic Acids Research* **32**, W400-W404 (2004).
4. Yildiz O, Vinothkumar KR, Goswami P, Kuhlbrandt W. Structure of the monomeric outer-membrane porin OmpG in the open and closed conformation. *EMBO J* **25**, 3702-3713 (2006).
5. Grosse W, *et al.* Structure-based engineering of a minimal porin reveals loop-independent channel closure. *Biochemistry* **53**, 4826-4838 (2014).
6. Subbarao GV, van den Berg B. Crystal structure of the monomeric porin OmpG. *J Mol Biol* **360**, 750-759 (2006).
7. Korkmaz-Ozkan F, Koster S, Kuhlbrandt W, Mantele W, Yildiz O. Correlation between the OmpG secondary structure and its pH-dependent alterations monitored by FTIR. *J Mol Biol* **401**, 56-67 (2010).
8. Grosse W, *et al.* Structural and functional characterization of a synthetically modified OmpG. *Bioorg Med Chem* **18**, 7716-7723 (2010).
9. Doreleijers JF, *et al.* NRG-CING: integrated validation reports of remediated experimental biomolecular NMR data and coordinates in wwPDB. *Nucleic Acids Research* **40**, D519-D524 (2012).
10. Laskowski RA, Rullmann JAC, MacArthur MW, Kaptein R, Thornton JM. AQUA and PROCHECK-NMR: Programs for checking the quality of protein structures solved by NMR. *Journal of Biomolecular Nmr* **8**, 477-486 (1996).
11. Vriend G. WHAT IF: a molecular modeling and drug design program. *J Mol Graph* **8**, 52-56, 29 (1990).
12. Liang B, Tamm LK. Structure of outer membrane protein G by solution NMR spectroscopy. *Proc Natl Acad Sci U S A* **104**, 16140-16145 (2007).
13. Cock PJ, *et al.* Biopython: freely available Python tools for computational molecular biology and bioinformatics. *Bioinformatics* **25**, 1422-1423 (2009).
